# Epidemiology report: trends in sex-specific cerebrovascular disease mortality in Europe based on WHO mortality data

**DOI:** 10.1093/eurheartj/ehy378

**Published:** 2018-08-14

**Authors:** Rushabh Shah, Elizabeth Wilkins, Melanie Nichols, Paul Kelly, Farah El-Sadi, F Lucy Wright, Nick Townsend

**Affiliations:** 1 Centre on Population Approaches for Non-Communicable Disease Prevention, Nuffield Department of Population Health, University of Oxford, Old Road Campus, Oxford, UK; 2 Global Obesity Centre, Faculty of Health, Deakin University, Geelong, VIC, Australia; 3 Physical Activity for Health Research Centre, Institute of Sport, Physical Education and Health Sciences, University of Edinburgh, Edinburgh, UK; 4 Cancer Epidemiology Unit, Nuffield Department of Population Health, University of Oxford, Old Road Campus, Oxford, UK; 5 Department for Health, University of Bath, Claverton Down, Bath, UK

**Keywords:** Stroke, Cerebrovascular disease, Mortality, Trends, Europe, Joinpoint regression analysis

## Abstract

**Aims:**

There have been substantial declines in cerebrovascular disease mortality across much of Europe, mirroring trends in deaths from cardiovascular disease as a whole. No study has investigated trends in cerebrovascular disease, and its subtypes within all European countries. This study aimed to examine sex-specific trends in cerebrovascular disease, and three of its sub-types: ischaemic stroke, haemorrhagic stroke, and subarachnoid haemorrhage (SAH), in Europe between 1980 and 2016.

**Methods and results:**

Sex-specific mortality data for each country of the World Health Organization (WHO) Europe region were extracted from the WHO global mortality database and analysed using Joinpoint software to examine trends. The number and location of significant joinpoints for each country by sex and subtype was determined using a log-linear model. The annual percentage change within each segment was calculated along with the average annual percentage change over the duration of all available data. The last 35 years have seen large overall declines in cerebrovascular disease mortality rates in the majority of European countries. While these declines have continued steadily in more than half of countries, this analysis has revealed evidence of recent plateauing and even increases in stroke mortality in a number of countries, in both sexes, and in all four geographical sub-regions of Europe. Analysis by stroke sub-type revealed that recent plateauing was most common for haemorraghic stroke and increases were most common for ischaemic stroke.

**Conclusion:**

These findings highlight the need for continued research into the inequalities in both current stroke mortality outcomes and trends across Europe, as well as the causes behind any recent plateauing of total cerebrovascular disease or its subtypes.

## Introduction

In recent decades, there have been substantial declines in cerebrovascular disease mortality across much of Europe, mirroring broader trends in deaths from cardiovascular disease (CVD) as a whole.^1^ These declines have been attributed to reduced disease incidence following improvements in primary prevention and risk factor management combined with improvements in treatment and care, leading to reduced case-fatality.^2^ Despite these decreases, cerebrovascular diseases remain the second largest single cause of death in Europe after ischaemic heart disease, responsible for 9% and 13% of all deaths in men and women, respectively.^1^ Moreover, recent upward trends in the prevalence of known risk factors such as obesity and diabetes, as well as plateauing population blood pressure levels,^1^ raise concerns about possible slow-downs or reversals in cerebrovascular disease mortality progress seen so far.

Evidence of recent plateauing in coronary heart disease (CHD) mortality rates has been demonstrated in at least one age group in 15 European Union (EU) countries for men and 12 countries for women in an analysis from 1980 to 2009.^3^ A recent analysis of trends in cerebrovascular disease mortality in the EU also revealed recent plateaus or increases in mortality in eight countries among males and seven among females.^4^ Existing trend studies of CVDs have tended to focus on broad categories of disease and have been restricted to EU member states.^3^^,^^4^ This study aims to examine the evidence for recent plateauing in sex-specific trends in cerebrovascular disease as a whole, and also for three cerebrovascular disease sub-types—ischaemic stroke, haemorrhagic stroke, and subarachnoid haemorrhage (SAH)—in Europe between 1980 and 2016.

## Methods

We extracted data on cause-specific mortality and population size by sex in 5-year age groups (up to and including 85 and over) from the World Health Organization (WHO) global mortality database for individual countries of the WHO European region (EUR). These data have been used widely in the analysis of CVD deaths within Europe^1^^,^^3^^,^^5–9^ including more specifically on stroke,^1^^,^^4^^,^^5^^,^^9^ as well as being used in analyses of mortality from other conditions within the continent.^10–12^ On top of this, WHO mortality data have been used in the analysis of mortality on a global level.^13–15^ The WHO database collates data on the absolute number of medically certified deaths from national authorities based on their vital registration systems. The data presented in the WHO Mortality Database are as submitted by individual countries to WHO.^1^^,^^5^^,^^8^ All analyses, interpretations, and conclusions are those of the authors, not the WHO, which is responsible only for the provision of the original data.

We (R.S., E.W. and N.T.) extracted data on the number of deaths from all cerebrovascular diseases, plus three cerebrovascular disease sub-types: ischaemic stroke, haemorrhagic stroke, and SAH, defined according to the following International Classification of Disease (ICD) codes: ICD-10 (International Classification of Diseases, tenth version) codes I60-I69 and ICD 9 codes 430-438, ICD 10 mortality tabulation list 1 codes 1069 and ICD 9A/9B, ICD 9N codes B290-B299 and ICD 08A codes A085. We computed age-standardized mortality rates (ASMRs) for each country, by sex, by standardizing aggregate rates to the European Standard Population (ESP), using the direct method.

We extracted and analysed data for the years 1980 (or the establishment of the present day country if later) to 2016 (or the most recently available year). The availability of data varied by country. Moreover, over the period studied, death registration or population data were missing for some years in some countries. We obtained deaths in Germany for the years prior to 1990 by combining death registration and population data for the former Democratic Republic of Germany and the former Federal Republic of Germany, then calculated overall rates. We analysed England, Scotland, Wales, and Northern Ireland together as the UK.

We performed Joinpoint regression analysis to identify periods with statistically distinct log linear trends in death rates from all cerebrovascular diseases and cerebrovascular disease sub-types over time within each sex and country. This analysis identifies inflexion points (or ‘joinpoints’) at which there is a significant change in trends using a series of permutation tests, with Bonferroni adjustment for multiple comparisons. We set the two-sided significance level at *P* < 0.05 for all tests. We determined the number and location of significant joinpoints for each country by sex using a log-linear model, and computed the annual percentage change (APC) within each segment. Use of a log-linear model enables the analysis of constant percentage (rather than absolute) change in the prevalence over time. We defined evidence to suggest a recent plateau in the mortality trend as the situation where the final joinpoint segment (in the best fitted model) was less steeply negative compared with the preceding segment. A negative slope was defined as one in which the APC was significantly negative, conferring a decreasing trend. A flat slope was defined as one in which the APC was not significantly different to zero. We defined evidence to suggest an increase in mortality as the situation where the APC was significantly positive, conferring an increasing trend. We also calculated the average annual percentage change (AAPC) for the overall period (1980–2016) for each country by sex. All definitions were designed a priori to agree with previous studies.^3^^,^^16^

We analysed trends by European geographical region, using the categories of Western Europe, Central Europe, Eastern Europe, and Central Asia, as defined by the Global Burden of Disease (GBD) study.^4^^,^^17^ All countries of WHO Europe fitted this categorisation, with the exception of Turkey that was found in the Middle East and North Africa region by GBD classification. We conducted all analyses using JoinPoint regression program version 4.4.0 (Statistical research and applications branch, National Cancer institute, USA). JoinPoint regression Program enables the user to test whether or not an apparent change in trend is statistically significant, by testing whether a multi-segmented line is a significantly better fit than a straight or less-segmented line. Line segments are joined at inflexion points called joinpoints. Each joinpoint denotes a statistically significant (*P* < 0.05) change in trend. Joinpoint fits the selected trend data into the simplest joinpoint model that the data allow. Since an additional joinpoint is only added to the model if the change in trend is statistically significant, each of the joinpoints displayed from the chosen model can be interpreted as a significant change in the trend. Once the line segments are established, the estimated annual percent change is used to describe and test the statistical significance of the trends in the model. Testing the hypothesis (two-sided *P*-value <0.05) that the annual percent change is equal to zero is equivalent to testing the hypothesis that the trend in the mortality rates is neither increasing nor decreasing.^18^^,^^19^ The JoinPoint regression programme has been used extensively in previous research on trends in CVD^6^^,^^16^^,^^20–23^ including cerebrovascular disease.^4^^,^^9^ We used Microsoft Excel 2016 for data management and manipulation, including the calculation of ASMRs, and as a data input file for JoinPoint.

## Results

Supplementary material online, *Table S1* provides an overview of the available years of data, the population size, and total number of deaths and stroke deaths by sex in 2016 (or the most recent year available) for 51 of the 53 countries of the European region. No data were available for Andorra or Monaco. Complete data for all 37 study years were available for five countries for all cerebrovascular diseases. Turkey had the lowest data availability, with available data from only 2009–2013 (5 years), followed by San Marino where data were available for 1995–2000 and 2005 (7 years).

**Table 1:** Summary of most recent trend in cerebrovascular disease and stroke sub-type mortality, by number and percentage of countries

	Cerebrovascular disease		Ischaemic stroke		Haemorrhagic stroke		Subarachnoid haemorrhage	
	Number	%	Number	%	Number	%	Number	%
Males
Significant decrease^a^	33	65	19	37	22	43	18	35
Final segment less steeply decreasing than previous segment^b^	7	14	3	6	3	6	3	6
No significant change	8	16	13	26	15	29	17	33
Final segment increasing^c^	3	6	8	16	3	6	5	10
No data	0	0	8	16	8	16	8	16
Females								
Significant decrease^a^	33	65	16	31	20	39	18	35
Final segment less steeply decreasing than previous segment^b^	6	12	4	8	8	16	2	4
No significant change	10	20	14	28	14	28	15	29
Final segment increasing^c^	2	4	9	18	1	2	8	16
No data	0	0	8	16	8	16	8	16

a Final segment significantly decreasing and more negative than previous segment or for trends with no joinpoints a significant decrease overall.

b Final segment significantly decreasing but less negative than previous segment.

c Final segment significantly increasing or no joinpoints or for trends with no joinpoints a significant increase overall.

In the latest available year, the number of stroke deaths was higher in females than males in 50 of the 51 countries with available data. The exception was Kyrgyzstan. The percentage of all deaths due to stroke was higher in females than males in all countries. However, ASMRs from stroke, which control for differences in population size and population age structure, were higher in males than females in all countries.

On average, the percentage of total deaths due to stroke in the latest available year was lowest in Western Europe (5.9% in males, 8.2% in females) and highest in Eastern Europe (11.6% in males, 17.5% in females). The lowest ASMRs for stroke were also found in Western Europe and the highest in Eastern Europe. In males, the ASMR for stroke ranged from 49 per 100 000 population in France to 131 in San Marino among Western European countries, from 110 in the Czech Republic to 391 in Bulgaria among Central European countries, from 82 in Estonia to 331 in Russia in Eastern European countries, and from 152 in Armenia to 345 in Azerbaijan in Central Asia. In females, the ASMR for stroke ranged from 41 in France to 112 in Greece among Western European countries, from 87 in Slovenia to 301 in Bulgaria among Central European countries, from 55/100 000 in Estonia to 253 in Moldova in Eastern European countries, and from 142 in Armenia to 342 in Azerbaijan in Central Asia (see Supplementary material online, *Table S1*).

### Average annual percentage changes: overall trends in mortality

Supplementary material online, *Tables S2*–*S5* summarize the AAPCs for mortality from all cerebrovascular diseases and three cerebrovascular disease sub-types: ischaemic stroke, haemorrhagic stroke, and SAH by sex. For cerebrovascular disease as a whole, significant decreases in ASMRs over the available years between 1980 and 2016 were seen in 34 countries among males (67%) and 34 countries among females (67%). The proportion of countries experiencing a significant decrease in cerebrovascular disease mortality was greatest in Western Europe in both males (96%) and females (91%). Among males, the smallest proportion of countries with a significant decrease was found in Central Asia (25%), whilst 62% of countries in Central Europe, and 43% of countries in Eastern Europe experienced significant declines. Among females, Central Asia had the smallest proportion of countries with a significant decrease (13%), compared with 57% in Eastern Europe and 69% in Central Europe.

**Figure 1:**
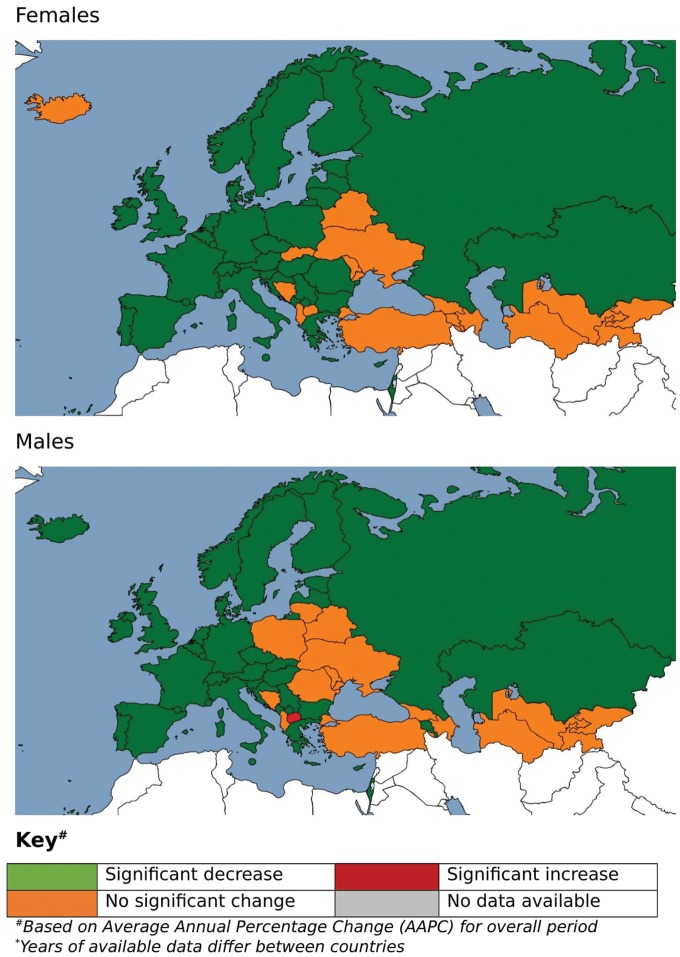
Map of trends in cerebrovascular age-standardized mortality rates, Europe 1980–2016 (years of available data differ between countries). ^a^Based on average annual percentage change (AAPC) for overall period.

The median AAPC (covering the full 37 year period) for all cerebrovascular diseases across all countries was −2.7% for males and −2.7% for females. In both sexes, the median AAPC was most negative in Western Europe (−3.6% for males, −3.6% for females) and least negative in Eastern Europe (−0.7%) for males, and in Central Asia for females (-0.9%). AAPCs in males in Central Asia (-1.0%) and Central Europe (−0.9%) were similar. In females the median AAPC in Central Europe (-1.7%) was more negative than for Eastern Europe (-1.0%). Despite these broad regional differences, the largest decreases occurred in countries spanning different regions: Austria, the Czech Republic, Estonia, Luxembourg, Montenegro, Portugal and Turkmenistan (AAPCs ≤−4.5% for both sexes). Males in Macedonia were the only to show a significant increase over the period of study (0.6%). However, the largest positive AAPCs amongst both males (9.7%) and females (3.3%) were found in San Marino over the seven data points available for the country, although neither were significant. (*Figure 1*, Supplementary material online, *Table S2*).

**Figure 2:**
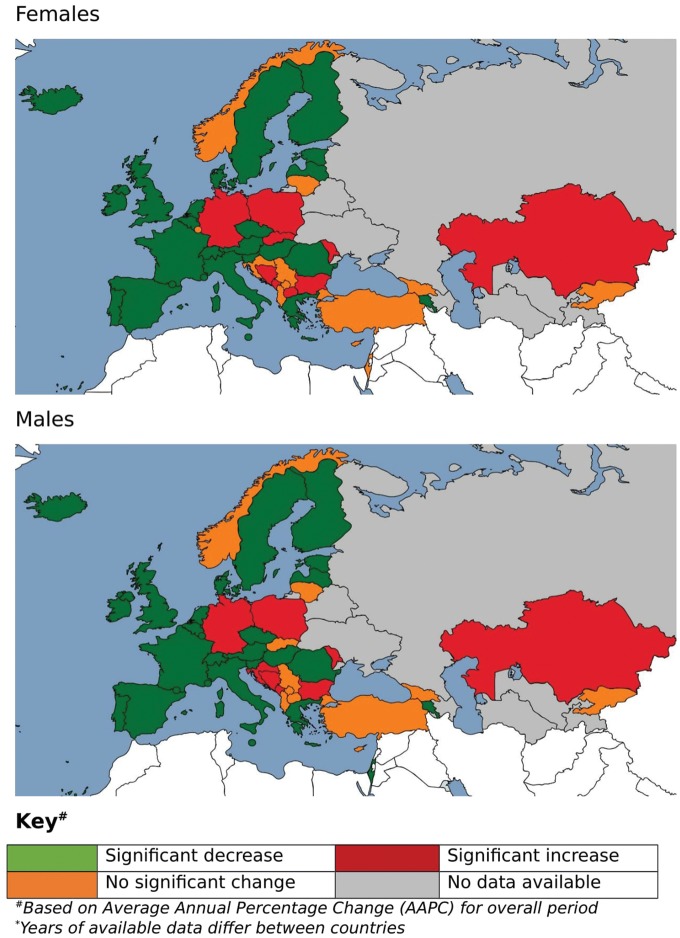
Map of trends in ischaemic stroke age-standardized mortality rates, Europe 1980–2016 (years of available data differ between countries). ^a^Based on average annual percentage change (AAPC) for overall period.

For the three stroke sub-types, data were available for only 43 countries, with most of the countries with missing data found in Eastern Europe and Central Asia. More than half of countries with available data experienced significant decreases in mortality from ischaemic stroke (56% for males, 51% for females) and haemorrhagic stroke (58% for males, 67% for females). In contrast, although 56% of countries showed decreases in ASMRs for SAH in males, only 42% did so for females. The median decrease for SAH (APC −1.9% for males, −1.2% for females) was lower than for ischaemic stroke (APC −2.2% for males, −2.2% for females) and haemorrhagic stroke (APC −2.0% for males, −2.5% for females), particularly among females (*Figure 2*, Supplementary material online, *Table S3*).

**Figure 3:**
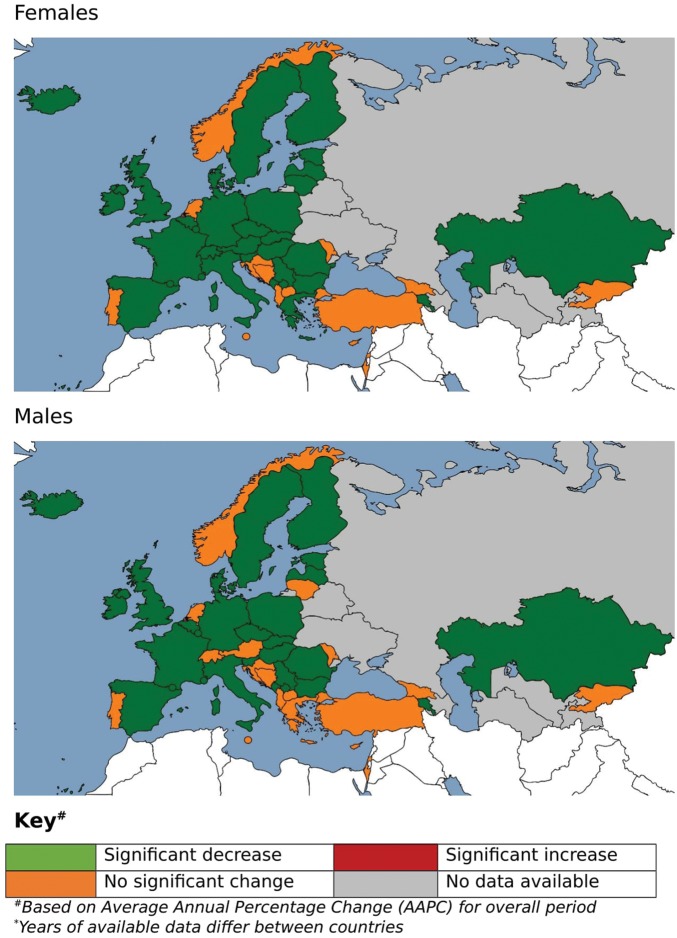
Map of trends in haemorrhagic stroke age-standardized mortality rates, Europe 1980–2016 (years of available data differ between countries). ^a^Based on average annual percentage change (AAPC) for overall period.

The median AAPC was negative in all regions for all three stroke sub-types, with the exception of ischaemic stroke in Central Asia (+4.5% for males, +3.8% for females) and Central Europe (+2.4% for males, +2.1% for females). The greatest median AAPC decreases for ischaemic stroke were found in Western Europe in males (−3.9%) and females (−3.7%). The greatest median decreases in SAH were found in Eastern Europe amongst males (−2.9%) and in Central Asia amongst females (−1.9%); the smallest median decreases were found in Central Europe for both sexes (−1.3% for males, −1.2% for females). Conversely, for haemorraghic stroke Western Europe showed the smallest median decreases (−1.5% for males, −2.2% for females) and Central Asia the greatest median decrease (−4.7% for males, −4.3% for females). There were significant mortality increases for ischaemic stroke in Germany, Bosnia, Bulgaria, Montenegro, Poland, Moldova and Kazakhstan for both males and females, Croatia for males only and Slovakia and TFYR of Macedonia for females only. No countries showed a significantly increasing trend for haemorraghic stroke, although a number of countries did for SAH: Romania for both sexes, Turkey for males only and Luxembourg, Bulgaria and Macedonia for females only (*Figure 3*, Supplementary material online, *Table S4*).

**Figure 4:**
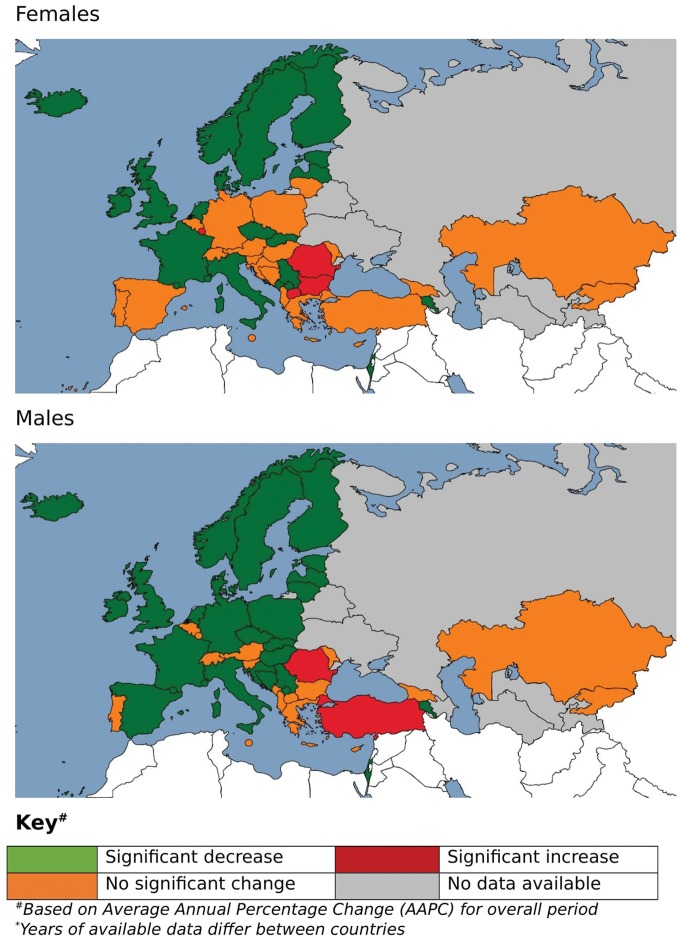
Map of trends in subarachnoid haemorrhage age-standardized mortality rates, Europe 1980–2016 (years of available data differ between countries). ^a^Based on average annual percentage change (AAPC) for overall period.

For the vast majority of countries, there was no significant difference in the AAPC between the sexes, with the exceptions of Bulgaria, Lithuania and Spain for all cerebrovascular disease; Estonia, Hungary, Italy, Lithuania, Poland, Serbia and Spain for haemorraghic stroke and Germany, Netherlands and Spain for SAH (*Figure 4*, Supplementary material online, *Table S5*).

### Identifying inflexion points and evidence for plateaus

**Figure 5:**
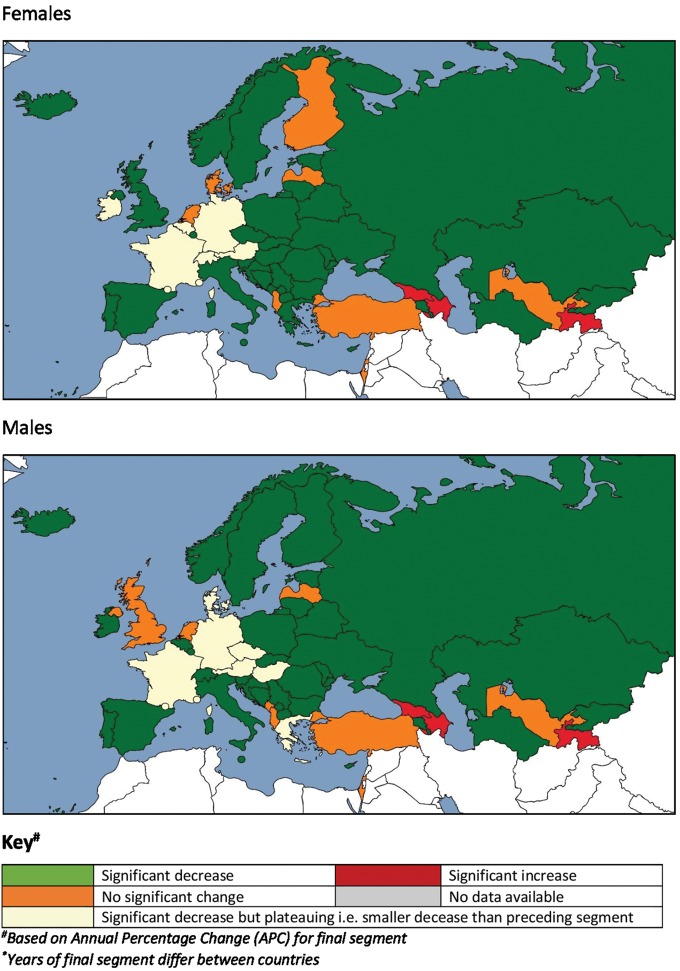
Map of most recent trend in cerebrovascular age-standardized mortality rates, Europe (years of final segment differ between countries). ^a^Based on annual percentage change (APC) for final segment.

For each sex for cerebrovascular disease as a whole, there was a mixture of countries demonstrating continuous linear trends (no joinpoints identified) and models containing one to five joinpoints over 30 years of analysis. Seventeen countries, predominantly in Central and Eastern Europe and Central Asia, had segments of significantly positive mortality trends in males, females or both sexes. There was a steeper decrease in the most recent trend segments than in the previous segment in more than half of countries (65% in both sexes). However, there was evidence of a recent plateau in mortality trends (where the rate of mortality decreases in the most recent period was less pronounced than in the previous period) in seven countries (14%) among males, and six countries (12%) among females. These countries spanned Western and Central Europe and included the Austria, France and Germany for both sexes, Denmark, Greece, Czech Republic and Hungary for males only, and Belgium, Ireland and Switzerland for females only. Eight countries (16%) showed a flat slope, or no significant change in the final segment in males, with ten countries (20%) doing so for females. There was also evidence of a recent increase in mortality trends in three countries (6%) for males (Azerbaijan, Georgia and Tajikistan) and two countries (4%) for females (Azerbaijan and Tajikistan) (*Figure 5*, Supplementary material online, *Table 1*).

The tendency for increases in mortality was greatest for ischaemic stroke with recent increases in eight countries (16%) for males and nine (18%) countries for females. This compared to five countries (10%) showing an increasing recent trend in SAH mortality amongst males and eight countries (16%) for females. Haemorraghic stroke demonstrated fewer increasing recent trends, with three countries (6%) for males and one country (2%) for females (*Table **1*).

## Discussion

The last 35 years have seen large overall declines in cerebrovascular disease mortality in the majority of European countries. While these declines have continued at a steady or increasing rate in more than half of countries, this analysis has revealed evidence of recent plateauing and even increases in stroke mortality in a number of individual countries. Recent mortality plateaus and increases have been observed in both sexes, and in countries spanning all four geographical sub-regions of Europe, although significant increases in stroke mortality appear to be particularly common in Central Asia.

There have also been overall decreases in mortality for three stroke sub-types over the past 35 years. These decreases have been greatest for ischaemic stroke and haemorrhagic stroke and smaller for SAH. As with cerebrovascular disease as a whole, recent mortality plateaus and increases have been seen for these sub-types in several geographically diverse individual countries. The tendency for recent plateauing was greatest for haemorraghic stroke, while recent increases in mortality were most common for ischaemic. Recent increases for all cerebrovascular disease also appear to be most common in Central Asia, and is slightly higher in males than females.

These findings of overall decreases in cerebrovascular disease mortality with emerging mortality plateaus and increases in several individual countries are consistent with those of Wang *et al.* for stroke,^4^ and with those of Nichols *et al.* for CHD.^3^ Both of these studies analysed trends up to 2009, whereas this study extends the analysis period to 2016. This study also analyses trends in Europe as a whole as opposed to EU member states only, and in doing so, highlights the tendency for recent increases in cerebrovascular disease mortality in Central Asian countries. Moreover, in analysing trends for stroke sub-types in addition to cerebrovascular disease more broadly, this study identifies recent mortality increases in ischaemic stroke as a particular concern.

One possible explanation for the attenuation of stroke mortality declines in a number of countries relates to plateauing or increases in stroke risk factors leading to changes in the incidence of the disease. Particularly noteworthy here are the considerable increases in the prevalence of overweight/obesity and diabetes over the last 30 years across Europe, as well as the recent stabilisation of smoking prevalence and blood cholesterol levels in many countries after steep declines.^1^ Recent decades have also seen limited improvements in case fatality rates for stroke, which could be another contributing factor.^24^ These most likely relate to improvements in treatment, although inequalities of this may exist between European countries.

This analysis has revealed differences in mortality trends across the three stroke sub-types, with recent plateauing most common for haemorraghic stroke and mortality increases most common for ischaemic stroke. Although trends in sub-types may be biased as data were predominantly missing from countries with higher mortality rates/different trends, these three sub-types have distinct pathophysiologies, and accordingly, the relative importance of different risk factors varies between them. The aforementioned plateauing in smoking prevalence could partly account for the different trends, with cigarette smoking a well-established risk factor for cerebral infarction^25–28^ and SAH,^29^ but less clearly so for intracerebral haemorrhage.^26^^,^^28^^,^^30^^,^^31^ Moreover, recent plateaus in mean blood cholesterol levels following sustained declines^1^ could play a role: cholesterol levels are positively associated with the risk of ischaemic stroke and SAH but inversely related to the risk of haemorrhagic stroke.^32^ Increased government-supported primary prevention efforts to tackle these risk factors should be a priority moving forwards, and will be most effective combined with improved treatment and long-term support to cover the whole stroke pathway. These efforts will be especially important in Central Asian countries, where there is most evidence of mortality increases.

The WHO mortality and population data are a valuable resource, permitting large-scale analysis, and cross-national comparisons such as those in this study. In general, these data are of high quality and are available for a large number of years for many countries. They have been used in a large number of studies on mortality within individual countries, across Europe and globally. That said, data coverage and quality is variable.

The data presented in the WHO Mortality Database, and in this article are as submitted by individual countries to WHO. No adjustments have been made to account for potential bias in reporting. As a result, the quality of mortality data varies between countries, with more accurate data for countries with well-functioning vital registration systems compared with those with weaker systems.^1^^,^^5^ We undertook no further data quality assessments than those already carried out by the WHO. They define data usability as the product of the proportion of deaths assigned to a set of ill‐defined (garbage) cause of death codes and 100 less the percentage of completeness.^26^ The WHO identified three countries from our sample as having data of lower usability (i.e. below 65%): Albania (60%), Georgia (61%), and Turkey (58%). Four countries were also identified as having a top of range fraction of garbage codes of more than 25% over all the years of data available: Bulgaria (28%), Greece (27%), Montenegro (29%), and Poland (31%). A number of countries also reported death registration data using a summarized cause list: Belarus, Ireland, Kazakhstan, Portugal, Russian Federation, The former Yugoslav Republic of Macedonia, Ukraine, and Uzbekistan.^26^ These data quality measures apply to a range of data rather than those pertaining to cerebrovascular disease specifically.^33^

Age-standardized rates can only be calculated where data on the absolute number of an outcome and the population are available in comparable age-specific aggregates. There was a complete absence of data for Andorra and Monaco (though these countries represent a small fraction of total European population), eight countries had no mortality data by stroke sub-type, and for others data were available for only a limited number of years over the study period. Central Asian and Eastern European countries were particularly affected, with sub-type data available for only three and four countries, respectively. This limits the power of the regional comparisons presented here, and highlights the importance of improved national stroke surveillance in these regions. There is also inter-country variability in ICD coding practices. For example, data from the WHO MONICA project suggest that official mortality statistics in France underreport CVD deaths compared with other countries, and that doctors in France are more likely to code CVD deaths as ‘other causes’, resulting in an underestimate of the true numbers and proportions of deaths from CVD.^34^ The necessary use in this historical trends study of three different ICD coding schemes is a further limitation, although more so for the sub-types analysis than the broader cerebrovascular disease category. As is the large proportion of stroke mortality that is unspecified and cannot therefore be included in sub-type analysis.

In addition to issues of data, there are limitations of the joinpoint approach to analysis. The method used to define a ‘recent plateau’ in trends provides a general indication at the European level of whether these is an emerging overall pattern of stabilising trends in stroke mortality rates. However, it is a simplified approach that may not give a holistic indication of a ‘recent plateau’ for a small number of subgroups or countries. This is particularly true in cases of brief trend segments. For example, both males and females in Hungary were classified as having ‘evidence of a recent plateau’ for ischaemic stroke as the most recent joinpoint segments had an APC of −4.6% and −4.1% respectively, while the preceding segments had an APC of −17.7% and −18.9%, respectively. In both cases, however, the penultimate segments only span three years (2003–2006), after which there has been a consistent annual percentage reduction in mortality rates.

A final limitation of this study is that it only seeks to describe trends in cerebrovascular mortality and does not aim to explain them. Further work could look to investigate the association between these mortality outcomes and sociodemographic characteristics, such as measures of income and health care expenditure. A weakness of the outcome data used here and many of these sociodemographic measures, is that they are collected and reported at the country level. Meaning that any study using them would prove ecological in nature. A richer dataset, arranged by regions within countries, would allow a more detailed analysis on within country and between country inequalities.

## Conclusion

Despite overall declines in stroke mortality in Europe over the past 35 years, there is emerging evidence of recent mortality plateaus and increases in a number of individual countries. Evidence of recent increases in stroke mortality is most common in Central Asia. Analysing by stroke sub-type reveals that recent plateauing is most common for haemorrhagic and increases are most common for ischaemic stroke. These findings highlight the need for continued research into the inequalities in both the stroke mortality outcomes and recent trends across Europe, as well as the causes behind any recent plateauing of total cerebrovascular disease or its subtypes. Continued monitoring of trends in cerebrovascular disease and sub-type mortality, incidence, case-fatality, and risk factors across the region is paramount to ensure interventions are well-targeted, and should be a priority for future research. Incorporating individual level data into country level analysis, through hierarchical or multilevel techniques, would be useful in examining within country trends and determinants. The collecting and reporting of these types of data could prove invaluable in the development of public health approaches to prevent stroke mortality.

## Funding

This work was supported by the British Heart Foundation coming under the BHF CVD epidemiology programme [006/P&C/CORE/2013/OXFSTATS to N.T.]; the European Heart Network (to E.W.); and an Alfred Deakin Postdoctoral Fellowship at Deakin University (to M.N.).

**Conflict of interest:** none declared.

## Supplementary Material

Supplementary Table S1

Supplementary Table S2

Supplementary Table S3

Supplementary Table S4

Supplementary Table S5
