# Supplementary material for: Epidemiology report: trends in sex-specific cerebrovascular disease mortality in Europe based on WHO mortality data
Source: Eur Heart J. 2018 Aug 14;40(9):755–64. doi: 10.1093/eurheartj/ehy378 (PMC6396027; doi:10.1093/eurheartj/ehy378)
Supplement: Supplementary Table S1 [file ehy378_supplementary_table_s1.docx]

**Supplementary Table 1: Overview of data availability and numbers of deaths and population in latest available year by country and sex**

|  | | **Males** | | | | | | **Females** | | | | | |
| --- | --- | --- | --- | --- | --- | --- | --- | --- | --- | --- | --- | --- | --- |
|  | | **Data years** | **Total population** | **Total deaths** | **Stroke deaths** | **Stroke deaths (% of total)** | **Stroke ASDR (per 100,000)** | **Data years** | **Total population** | **Total deaths** | **Stroke deaths** | **Stroke deaths (% of total)** | **Stroke ASDR (per 100,000)** |
| Austria | | 1980-2014 | 4,176,550 | 37,424 | 2,561 | 6.84 | 61 | 1980-2014 | 4,367,382 | 40,828 | 4,860 | 11.90 | 56 |
| Belgium | | 1980-2012 | 5,451,780 | 53,830 | 2,832 | 5.26 | 71 | 1980-2012 | 5,643,070 | 55,246 | 4,177 | 7.56 | 62 |
| Cyprus | | 1999-2000, 2004-2012 | 420,010 | 2,888 | 174 | 6.02 | 79 | 1999-2000, 2004-2012 | 443,932 | 2,384 | 226 | 9.48 | 78 |
| Denmark | | 1980-2012 | 2,771,208 | 25,458 | 1,499 | 5.89 | 92 | 1980-2012 | 2,815,877 | 25,535 | 1,847 | 7.23 | 64 |
| Finland | | 1980-2013 | 2,673,493 | 25,864 | 1,843 | 7.13 | 95 | 1980-2013 | 2,765,479 | 26,545 | 2,576 | 9.70 | 76 |
| France | | 1980-2011 | 30,780,362 | 279,954 | 13,386 | 4.78 | 57 | 1980-2011 | 32,738,715 | 276,077 | 18,377 | 6.66 | 44 |
| Germany | | 1990-2013 | 39,469,105 | 429,645 | 23,167 | 5.39 | 72 | 1990-2013 | 41,176,500 | 464,180 | 35,389 | 7.62 | 64 |
| Greece | | 1980-2012 | 5,431,556 | 60,135 | 6,674 | 11.10 | 203 | 1980-2012 | 5,661,227 | 56,533 | 9,194 | 16.26 | 136 |
| Iceland | | 1981-2009 | 161,548 | 1,049 | 75 | 7.15 | 88 | 1981-2009 | 157,698 | 999 | 84 | 8.41 | 72 |
| Ireland | | 1980-2009, 2011-2012 | 2,269,612 | 14,958 | 773 | 5.17 | 72 | 1980-2009, 2011-2012 | 2,315,795 | 14,546 | 1,162 | 7.99 | 72 |
| Israel | | 1980-2013 | 4,070,269 | 21,024 | 1,064 | 5.06 | 55 | 1980-2013 | 4,145,398 | 21,146 | 1,265 | 5.98 | 46 |
| Italy | | 1980-2003, 2004-2005 | 28,808,103 | 295,820 | 23,950 | 8.10 | 96 | 1980-2003, 2004-2005 | 30,731,623 | 317,687 | 37,303 | 11.74 | 83 |
| Luxembourg | | 1980-2014 | 278,544 | 1,919 | 85 | 4.43 | 53 | 1980-2014 | 277,775 | 1,786 | 139 | 7.78 | 52 |
| Malta | | 1980-2014 | 213,607 | 1,655 | 116 | 7.01 | 84 | 1980-2014 | 213,814 | 1,615 | 160 | 9.91 | 78 |
| Netherlands | | 1980-2014 | 8,320,862 | 67,121 | 3,718 | 5.54 | 68 | 1980-2014 | 8,483,570 | 72,102 | 5,618 | 7.79 | 64 |
| Norway | | 1980-2014 | 2,583,129 | 19,685 | 1,114 | 5.66 | 66 | 1980-2014 | 2,554,300 | 20,659 | 1,591 | 7.70 | 59 |
| Portugal | | 1980-2003, 2007-2014 | 4,976,859 | 53,484 | 5,362 | 10.03 | 134 | 1980-2003, 2007-2014 | 5,480,437 | 51,720 | 6,910 | 13.36 | 102 |
| San Marino | | 1995-2000, 2005 | 14,637 | 105 | 9 | 8.57 | 109 | 1995-2000, 2005 | 15,205 | 83 | 7 | 8.43 | 55 |
| Spain | | 1980-2014 | 22,840,092 | 201,571 | 11,573 | 5.74 | 62 | 1980-2014 | 23,615,035 | 194,259 | 16,006 | 8.24 | 52 |
| Sweden | | 1980-2014 | 4,843,299 | 43,440 | 2,857 | 6.58 | 73 | 1980-2014 | 4,852,811 | 45,622 | 3,731 | 8.18 | 62 |
| Switzerland | | 1980-2013 | 3,995,308 | 31,257 | 1,465 | 4.69 | 51 | 1980-2013 | 4,094,038 | 33,704 | 2,238 | 6.64 | 47 |
| United K. | | 1980-2013 | 31,532,873 | 279,171 | 16,260 | 5.82 | 69 | 1980-2013 | 32,572,781 | 297,287 | 16,197 | 5.45 | 47 |
| **Western Europe** | |  | 206,082,806 | 1,947,457 | 120,557 | 6.19 |  |  | 215,122,462 | 2,020,543 | 169,057 | 8.37 |  |
| Albania | | 1987-1989, 1992-2009 | 1,463,539 | 6,551 | 1,532 | 23.39 | 284 | 1987-1989, 1992-2009 | 1,463,983 | 5,588 | 1,840 | 32.93 | 345 |
| Bosnia | | 1985-1991, 2011 | 1,877,910 | 18,360 | 2,050 | 11.17 | 216 | 1985-1991, 2011 | 1,958,467 | 17,620 | 2,599 | 14.75 | 199 |
| Bulgaria | | 1980-2012 | 3,555,920 | 54,827 | 9,964 | 18.17 | 382 | 1980-2012 | 3,749,968 | 49,518 | 11,819 | 23.87 | 302 |
| Croatia | | 1985-2013 | 2,053,788 | 24,965 | 3,026 | 12.12 | 218 | 1985-2013 | 2,201,901 | 25,873 | 4,217 | 16.30 | 175 |
| Czech R. | | 1986-2013 | 5,161,617 | 53,740 | 4,249 | 7.91 | 136 | 1986-2013 | 5,349,102 | 51,925 | 6,067 | 11.68 | 112 |
| Hungary | | 1980-2013 | 4,699,585 | 61,986 | 5,268 | 8.50 | 173 | 1980-2013 | 5,166,883 | 64,315 | 7,121 | 11.07 | 128 |
| Montenegro | | 2000-2009 | 311,258 | 3,012 | 203 | 6.74 | 126 | 2000-2009 | 320,278 | 2,854 | 317 | 11.11 | 147 |
| Poland | | 1980-1996, 1999-2014 | 18,397,470 | 195,791 | 13,365 | 6.83 | 117 | 1980-1996, 1999-2014 | 19,608,684 | 180,676 | 17,644 | 9.77 | 91 |
| Romania | | 1980-2014 | 10,885,054 | 133,056 | 19,867 | 14.93 | 297 | 1980-2014 | 11,414,676 | 121,735 | 24,815 | 20.38 | 241 |
| Serbia | | 1998-2014 | 3,472,746 | 50,984 | 5,477 | 10.74 | 210 | 1998-2014 | 3,659,041 | 50,225 | 6,842 | 13.62 | 187 |
| Slovakia | | 1992-2010, 2011-2014 | 2,640,694 | 26,498 | 2,358 | 8.90 | 164 | 1992-2010, 2011-2014 | 2,777,955 | 24,847 | 2,829 | 11.39 | 132 |
| Slovenia | | 1985-2013 | 1,021,419 | 9,555 | 735 | 7.69 | 109 | 1985-2013 | 1,040,204 | 9,779 | 706 | 7.22 | 60 |
| Macedonia | | 1991-2013 | 1,033,990 | 10,136 | 1,746 | 17.23 | 363 | 1991-2013 | 1,030,042 | 9,072 | 1,855 | 20.45 | 298 |
| **Central Europe** | |  | **56,574,990** | **649,461** | **69,840** | **10.75** |  |  | **59,741,184** | **614,027** | **88,671** | **14.44** |  |
| Belarus | | 1981-1982, 1985-2003, 2007-2009, 2011 | 4,403,227 | 61,249 | 6,853 | 11.19 | 280 | 1981-1982, 1985-2003, 2007-2009, 2011 | 5,069,945 | 60,256 | 8,832 | 14.66 | 184 |
| Estonia | | 1981-1982, 1985-2012 | 614,654 | 7,460 | 416 | 5.58 | 109 | 1981-1982, 1985-2012 | 699,891 | 8,004 | 633 | 7.91 | 75 |
| Georgia | | 1981-1982, 1985-1992, 1994-2001, 2004-2007, 2009-2014 | 1,776,700 | 24,702 | 2,591 | 10.49 | 244 | 1981-1982, 1985-1992, 1994-2001, 2004-2007, 2009-2014 | 1,950,300 | 24,178 | 3,152 | 13.04 | 175 |
| Latvia | | 1980-2014 | 914,126 | 13,557 | 1,717 | 12.67 | 283 | 1980-2014 | 1,079,656 | 14,643 | 3,157 | 21.56 | 240 |
| Lithuania | | 1981-1982, 1986-2014 | 1,351,126 | 20,110 | 1,973 | 9.81 | 219 | 1981-1982, 1986-2014 | 1,581,241 | 20,142 | 3,537 | 17.56 | 179 |
| Moldova | | 1981-1982, 1985-2014 | 1,710,875 | 20,606 | 2,701 | 13.11 | 354 | 1981-1982, 1985-2014 | 1,845,522 | 18,888 | 3,293 | 17.43 | 279 |
| Russia | | 1980-2011 | 66,113,269 | 991,607 | 128,534 | 12.96 | 401 | 1980-2011 | 76,847,639 | 926,577 | 204,070 | 22.02 | 354 |
| Ukraine | | 1980-1982, 1985-2012, 2014 | 19,815,981 | 310,563 | 35,463 | 11.42 | 305 | 1980-1982, 1985-2012, 2014 | 23,015,591 | 321,598 | 52,423 | 16.30 | 231 |
| **Eastern Europe** |  | | **96,699,958** | **1,449,854** | **180,248** | **12.43** |  |  | **112,089,785** | **1,394,286** | **279,097** | **20.02** |  |
| Armenia | | 1981-1982, 1985-2003, 2006, 2012-2013 | 1,447,572 | 14,219 | 1,110 | 7.81 | 167 | 1981-1982, 1985-2003, 2006, 2012-2013 | 1,574,407 | 13,495 | 1,444 | 10.70 | 154 |
| Azerbaijan | | 1981-1982, 1985-2004, 2007 | 4,231,550 | 26,517 | 3,959 | 14.93 | 345 | 1981-1982, 1985-2004, 2007 | 4,349,750 | 23,922 | 5,130 | 21.44 | 342 |
| Kazakhstan | | 1981-1982, 1985-2003, 2005-2014 | 8,349,508 | 71,812 | 6,075 | 8.46 | 245 | 1981-1982, 1985-2003, 2005-2014 | 8,939,602 | 59,947 | 6,882 | 11.48 | 183 |
| Kyrgyzstan | | 1981-1982, 1985-2014 | 2,886,758 | 20,006 | 2,486 | 12.43 | 365 | 1981-1982, 1985-2014 | 2,949,058 | 15,545 | 2,334 | 15.01 | 268 |
| Tajikistan | | 1981-1982, 1985-2005 | 3,365,833 | 15,830 | 1,075 | 6.79 | 215 | 1981-1982, 1985-2005 | 3,344,329 | 13,081 | 1,193 | 9.12 | 165 |
| Turkmenistan | | 1981-1982, 1985-1998 | 2,337,800 | 18,079 | 626 | 3.46 | 156 | 1981-1982, 1985-1998 | 2,370,000 | 13,672 | 750 | 5.49 | 145 |
| Uzbekistan | | 1981-1982, 1985-2005, 2009-2014 | 13,069,360 | 81,336 | 7,236 | 8.90 | 1,492 | 1981-1982, 1985-2005, 2009-2014 | 13,097,660 | 68,257 | 8,746 | 12.81 | 1225 |
| **Central Asia** |  | | **35,688,381** | **247,799** | **22,567** | **9.11** |  |  | **36,624,806** | **207,919** | **26,479** | **12.74** |  |
| Turkey | 2009-2013 | | 38,164,870 | 196,709 | 16,167 | 8.22 | 140 |  | 37,889,747 | 160,824 | 19,704 | 12.25 | 124 |
| **Europe total** |  | |  | **4,491,280** | **409,379** |  |  |  |  | **4,397,599** | **583,008** |  |  |
