# Supplementary material for: Epidemiology report: trends in sex-specific cerebrovascular disease mortality in Europe based on WHO mortality data
Source: Eur Heart J. 2018 Aug 14;40(9):755–64. doi: 10.1093/eurheartj/ehy378 (PMC6396027; doi:10.1093/eurheartj/ehy378)
Supplement: Supplementary Table S2 [file ehy378_supplementary_table_s2.docx]

**Supplementary Table 2: JoinPoint analysis of trends in age standardised mortality rates for all cerebrovascular disease by country, geographic region and sex**

**Males**

|  | **Total study period** | **Period 1** | | **Period 2** | | **Period 3** | | **Period 4** | | **Period 5** | | **Period 6** | | |
| --- | --- | --- | --- | --- | --- | --- | --- | --- | --- | --- | --- | --- | --- | --- |
|  | **Average APC (%)^a^** | **Years** | **APC (%)** | **Years** | **APC (%)** | **Years** | **APC (%)** | **Years** | **APC (%)** | **Years** | **APC (%)** | **Years** | **APC (%)** |  |
| **Western Europe** | **-3.6** |  |  |  |  |  |  |  |  |  |  |  |  |  |
| Austria | -4.8* | 1980-1986 | -1.5 | 1986-1989 | -7.9 | 1989-2002 | -3.8* | 2002-2005 | -14.8* | 2005-2016 | -4.0* |  |  |  |
| Belgium | -3.5* | 1980-1983 | 1.2 | 1983-1987 | -8.2* | 1987-1993 | -0.8 | 1993-2015 | -4.0* |  |  |  |  |  |
| Cyprus | -4.0* | 2004-2016 | -4.0* |  |  |  |  |  |  |  |  |  |  |  |
| Denmark | -2.6* | 1980-1987 | -2.5* | 1987-1993 | 1.2 | 1993-2000 | -3.2* | 2000-2003 | 3.8 | 2003-2011 | -6.9* | 2011-2015 | -2.8* |  |
| Finland | -3.0* | 1980-1991 | -1.2* | 1991-2014 | -3.8* |  |  |  |  |  |  |  |  |  |
| France | -4.3* | 1980-1986 | -3.2* | 1986-1989 | -9.8* | 1989-2003 | -3.3* | 2003-2006 | -6.5* | 2006-2014 | -3.8* |  |  |  |
| Germany | -4.1* | 1980-1987 | -3.5* | 1987-1995 | -1.8* | 1995-2009 | -6.1* | 2009-2015 | -2.9* |  |  |  |  |  |
| Greece | -2.7* | 1980-2002 | -1.0* | 2002-2010 | -6.3* | 2010-2015 | -3.8* |  |  |  |  |  |  |  |
| Iceland | -2.7* | 1980-1998 | -0.7 | 1998-2016 | -4.7* |  |  |  |  |  |  |  |  |  |
| Ireland | -4.0* | 1980-2000 | -3.3* | 2000-2013 | -5.0* |  |  |  |  |  |  |  |  |  |
| Israel | -3.7* | 1980-1988 | -4.7* | 1988-1996 | 0.2 | 1996-1999 | -15.8 | 1999-2002 | 4.5 | 2002-2009 | -7.1* | 2009-2015 | -0.6 |  |
| Italy | -3.2* | 1980-1985 | 0.4 | 1985-2015 | -3.7* |  |  |  |  |  |  |  |  |  |
| Luxembourg | -5.9* | 1980-2010 | -5.1* | 2010-2015 | -10.4* |  |  |  |  |  |  |  |  |  |
| Malta | -3.1* | 1980-2015 | -3.1* |  |  |  |  |  |  |  |  |  |  |  |
| Netherlands | -2.8* | 1980-1997 | -2.3* | 1987-1991 | -0.2 | 1991-2002 | -2.3* | 2002-2011 | -6.8* | 2011-2014 | 2.9 | 2014-2016 | -2.2 |  |
| Norway | -3.5* | 1980-1996 | -1.7* | 1996-2015 | -4.9* |  |  |  |  |  |  |  |  |  |
| Portugal | -4.5* | 1980-1994 | -2.1* | 1994-2014 | -6.1* |  |  |  |  |  |  |  |  |  |
| San Marino | 9.7 | 1995-2005 | 9.7 |  |  |  |  |  |  |  |  |  |  |  |
| Spain | -4.4* | 1980-1999 | -3.9* | 1999-2015 | -5.0* |  |  |  |  |  |  |  |  |  |
| Sweden | -2.4* | 1980-1999 | -1.0* | 1999-2015 | -4.1* |  |  |  |  |  |  |  |  |  |
| Switzerland | -4.1* | 1980-2013 | -4.1* |  |  |  |  |  |  |  |  |  |  |  |
| United K. | -3.6* | 1980-1998 | -2.9* | 1998-2002 | 0.1 | 2002-2012 | -6.7* | 2012-2015 | -2.7 |  |  |  |  |  |
| **Central Europe** | **-0.9** |  |  |  |  |  |  |  |  |  |  |  |  |  |
| Albania | -0.9 | 1987-1994 | -3.0 | 1994-1997 | 18.4 | 1997-2010 | -3.8* |  |  |  |  |  |  |  |
| Bosnia | 0.6 | 1985-1990 | 8.4* | 1990-2014 | -1.0* |  |  |  |  |  |  |  |  |  |
| Bulgaria | -0.9* | 1980-2014 | -0.9* |  |  |  |  |  |  |  |  |  |  |  |
| Croatia | -2.2* | 1985-1994 | -1.2* | 1994-1998 | 3.7 | 1998-2016 | -3.9* |  |  |  |  |  |  |  |
| Czech R. | -4.8* | 1986-1990 | -1.9 | 1990-1997 | -5.8* | 1997-2003 | 0.1 | 2003-2007 | -10.4* | 2007-2016 | -6.0* |  |  |  |
| Hungary | -2.8* | 1980-2003 | -1.6* | 2003-2006 | -8.7 | 2006-2016 | -3.7* |  |  |  |  |  |  |  |
| Montenegro | -5.5* | 2000-2007 | -0.9 | 2007-2009 | -19.8 |  |  |  |  |  |  |  |  |  |
| Poland | -0.5 | 1980-1983 | -5.6* | 1983-1986 | 6.5 | 1986-1995 | 1.0* | 1995-2000 | 8.3* | 2000-2015 | -4.5* |  |  |  |
| Romania | -0.6 | 1980-1991 | 0.6 | 1991-1994 | 10.1 | 1994-2005 | -1.3* | 2005-2016 | -3.8* |  |  |  |  |  |
| Serbia | -3.1* | 1998-2007 | -1.4* | 2007-2015 | -4.9* |  |  |  |  |  |  |  |  |  |
| Slovakia | -0.8* | 1992-2014 | -0.8* |  |  |  |  |  |  |  |  |  |  |  |
| Slovenia | -3.5* | 1985-1992 | -0.9 | 1992-2003 | -4.0* | 2003-2006 | -10.8 | 2006-2009 | 4.7 | 2009-2015 | -5.6* |  |  |  |
| TFYR Macedonia | 0.6* | 1991-1998 | 3.4* | 1998-2013 | -0.6* |  |  |  |  |  |  |  |  |  |
| **Eastern Europe** | **-0.7** |  |  |  |  |  |  |  |  |  |  |  |  |  |
| Belarus | -0.4 | 1981-1987 | 2.2* | 1987-1991 | -1.5 | 1991-1999 | 3.0* | 1999-2010 | -1.8* | 2010-2014 | -6.3* |  |  |  |
| Estonia | -4.9* | 1981-1993 | -0.3 | 1993-2003 | -3.4* | 2003-2015 | -10.6* |  |  |  |  |  |  |  |
| Latvia | -1.6* | 1980-2003 | -0.3 | 2003-2009 | -7.4* | 2009-2015 | -0.7 |  |  |  |  |  |  |  |
| Lithuania | -0.1 | 1981-2006 | 0.7* | 2006-2016 | -1.9* |  |  |  |  |  |  |  |  |  |
| Republic of Moldova | -0.6 | 1981-1985 | 4.7 | 1985-1990 | -7.0* | 1990-2003 | 2.8* | 2003-2016 | -3.0* |  |  |  |  |  |
| Russia | -1.4* | 1980-1985 | 1.4 | 1985-1991 | -1.6* | 1991-1994 | 6.6 | 1994-1997 | -3.4 | 1997-2004 | 2.3* | 2004-2015 | -6.2* |  |
| Ukraine | -0.7 | 1981-1985 | 4.3 | 1985-1990 | -1.5 | 1990-1995 | 3.2* | 1995-1998 | -5.5 | 1998-2006 | -0.6 | 2006-2015 | -3.0* |  |
| **Central Asia** | **-1.0** |  |  |  |  |  |  |  |  |  |  |  |  |  |
| Armenia | -1.6* | 1981-1993 | 2.4* | 1993-2000 | -7.2* | 2000-2003 | 27.8* | 2003-2016 | -7.9* |  |  |  |  |  |
| Azerbaijan | -0.0 | 1981-1987 | 1.5 | 1987-1990 | -14.9* | 1990-1993 | 4.4 | 1993-2001 | -2.3* | 2001-2007 | 7.8* |  |  |  |
| Georgia | -1.6 | 1981-2007 | -0.7 | 2007-2012 | -18.9* | 2012-2015 | 24.9* |  |  |  |  |  |  |  |
| Kazakhstan | -1.4* | 1981-1998 | 2.0* | 1998-2007 | -2.3* | 2007-2015 | -7.3* |  |  |  |  |  |  |  |
| Kyrgyzstan | -0.5 | 1981-1995 | 1.7* | 1995-1998 | -3.7 | 1998-2006 | 1.3 | 2006-2015 | -4.3* |  |  |  |  |  |
| Tajikistan | -0.2 | 1981-1994 | 0.6 | 1994-1998 | -18.2* | 1998-2004 | 12.2* |  |  |  |  |  |  |  |
| Turkmenistan | -6.1 | 1981-1987 | 0.6 | 1987-1990 | -20.0 | 1990-1994 | 10.0 | 1994-1998 | -18.6* |  |  |  |  |  |
| Uzbekistan | 0.2 | 1981-1995 | 1.8* | 1995-2005 | -2.0 |  |  |  |  |  |  |  |  |  |
| **Middle East and North Africa** |  |  |  |  |  |  |  |  |  |  |  |  |  |  |
| Turkey | 0.2 | 2009-2015 | 0.2 |  |  |  |  |  |  |  |  |  |  |  |

**Females**

|  | **Total study period** | **Period 1** | | **Period 2** | | **Period 3** | | **Period 4** | | **Period 5** | | **Period 6** | |
| --- | --- | --- | --- | --- | --- | --- | --- | --- | --- | --- | --- | --- | --- |
|  | **Average APC (%)^a^** | **Years** | **APC (%)** | **Years** | **APC (%)** | **Years** | **APC (%)** | **Years** | **APC (%)** | **Years** | **APC (%)** | **Years** | **APC (%)** |
| **Western Europe** | **-3.6** |  |  |  |  |  |  |  |  |  |  |  |  |
| Austria | -4.6* | 1980-1986 | -1.4 | 1986-1989 | -8.5 | 1989-2002 | -3.5* | 2002-2005 | -15.3* | 2005-2016 | -3.5* |  |  |
| Belgium | -3.4* | 1980-1983 | 1.0 | 1983-1986 | -8.0* | 1986-2004 | -2.9* | 2004-2007 | -6.2 | 2007-2015 | -3.4* |  |  |
| Cyprus | -3.4* | 2004-2016 | -3.4* |  |  |  |  |  |  |  |  |  |  |
| Denmark | -2.4* | 1980-1989 | -1.3* | 1989-1993 | 1.3 | 1993-1998 | -3.7* | 1998-2003 | 0.2 | 2003-2013 | -6.0* | 2013-2015 | 1.4 |
| Finland | -3.0* | 1980-1993 | -1.4* | 1993-2008 | -4.7* | 2008-2014 | -2.0 |  |  |  |  |  |  |
| France | -4.1* | 1980-1986 | -3.4* | 1986-1989 | -8.4* | 1989-1994 | -4.4* | 1994-2003 | -2.8* | 2003-2007 | -6.5* | 2007-2014 | -2.9* |
| Germany | -4.0* | 1980-1988 | -3.8* | 1988-1993 | -0.9 | 1993-2008 | -5.1* | 2008-2015 | -3.9* |  |  |  |  |
| Greece | -3.0* | 1980-2004 | -1.1* | 2004-2015 | -7.2* |  |  |  |  |  |  |  |  |
| Iceland | -2.7 | 1980-1983 | -10.0 | 1983-1986 | 12.3 | 1986-1992 | -5.7* | 1992-1995 | 9.2 | 1995-2016 | -4.4* |  |  |
| Ireland | -3.8* | 1980-2001 | -3.6* | 2001-2005 | -7.5* | 2005-2013 | -2.5* |  |  |  |  |  |  |
| Israel | -4.2* | 1980-1996 | -3.8* | 1996-1999 | -12.1 | 1999-2005 | -0.5 | 2005-2008 | -11.0 | 2008-2015 | -1.8 |  |  |
| Italy | -3.0* | 1980-1985 | 0.3 | 1985-2015 | -3.5* |  |  |  |  |  |  |  |  |
| Luxembourg | -5.6* | 1980-1990 | -3.2* | 1990-1997 | -8.1* | 1997-2001 | -0.4 | 2001-2010 | -5.5* | 2010-2015 | -10.5* |  |  |
| Malta | -4.4* | 1980-1984 | -23.7* | 1984-1987 | 22.4 | 1987-2015 | -3.8* |  |  |  |  |  |  |
| Netherlands | -2.6* | 1980-1987 | -2.6* | 1987-1992 | 0.0 | 1992-2002 | -2.2* | 2002-2011 | -5.7* | 2011-2014 | 2.4 | 2014-2016 | -3.5 |
| Norway | -3.5* | 1980-1996 | -2.1* | 1996-2015 | -4.6* |  |  |  |  |  |  |  |  |
| Portugal | -4.6* | 1980-1996 | -2.4* | 1996-2014 | -6.5* |  |  |  |  |  |  |  |  |
| San Marino | 3.3 | 1995-2005 | 3.3 |  |  |  |  |  |  |  |  |  |  |
| Spain | -4.7* | 1980-1999 | -4.2* | 1999-2015 | -5.4* |  |  |  |  |  |  |  |  |
| Sweden | -2.7* | 1980-2002 | -1.4* | 2002-2005 | -6.9* | 2005-2012 | -3.0* | 2012-2015 | -6.9* |  |  |  |  |
| Switzerland | -4.1* | 1980-1985 | -5.8* | 1985-2013 | -3.8* |  |  |  |  |  |  |  |  |
| United K. | -3.6* | 1980-1986 | -1.6* | 1986-1998 | -3.3* | 1998-2002 | 0.7 | 2002-2015 | -5.9* |  |  |  |  |
| **Central Europe** | **-1.7** |  |  |  |  |  |  |  |  |  |  |  |  |
| Albania | 0.2 | 1987-2004 | 2.0* | 2004-2010 | -4.9 |  |  |  |  |  |  |  |  |
| Bosnia | 0.2 | 1985-1990 | 7.8* | 1990-2014 | -1.3* |  |  |  |  |  |  |  |  |
| Bulgaria | -1.3* | 1980-2014 | -1.3* |  |  |  |  |  |  |  |  |  |  |
| Croatia | -2.3* | 1985-1994 | -1.8* | 1994-1997 | 6.5 | 1997-2016 | -3.9* |  |  |  |  |  |  |
| Czech R. | -5.0* | 1986-1997 | -4.6* | 1997-2002 | 0.5 | 2002-2016 | -7.2* |  |  |  |  |  |  |
| Hungary | -3.3* | 1980-2003 | -2.1* | 2003-2006 | -9.7* | 2006-2014 | -2.9* | 2014-2016 | -9.2* |  |  |  |  |
| Montenegro | -4.5* | 2000-2006 | 0.1 | 2006-2009 | -13.0* |  |  |  |  |  |  |  |  |
| Poland | -1.0* | 1980-1982 | -6.0 | 1982-1995 | 1.1* | 1995-2000 | 8.3* | 2000-2015 | -4.9* |  |  |  |  |
| Romania | -1.1* | 1980-1988 | 1.5* | 1988-1991 | -4.1 | 1991-1994 | 9.3* | 1994-2005 | -1.4* | 2005-2016 | -4.5* |  |  |
| Serbia | -3.4* | 1998-2008 | -1.5* | 2008-2015 | -6.1* |  |  |  |  |  |  |  |  |
| Slovakia | -1.7 | 1992-2006 | -2.0* | 2006-2009 | 8.1 | 2009-2014 | -6.5* |  |  |  |  |  |  |
| Slovenia | -3.5* | 1985-2003 | -3.4* | 2003-2006 | -12.0 | 2006-2009 | 6.2 | 2009-2015 | -4.1* |  |  |  |  |
| TFYR Macedonia | 0.4 | 1991-2005 | 2.1* | 2005-2013 | -2.5* |  |  |  |  |  |  |  |  |
| **Eastern Europe** | **-1.0** |  |  |  |  |  |  |  |  |  |  |  |  |
| Belarus | -1.0 | 1981-1988 | 2.9* | 1988-1991 | -4.1 | 1991-2001 | 1.5* | 2001-2014 | -4.2* |  |  |  |  |
| Estonia | -5.7* | 1981-1993 | -0.8 | 1993-2003 | -3.9* | 2003-2015 | -11.7* |  |  |  |  |  |  |
| Latvia | -1.6* | 1980-1983 | 4.5 | 1983-2003 | -1.1* | 2003-2010 | -7.5* | 2010-2015 | 1.7 |  |  |  |  |
| Lithuania | -0.5* | 1981-2002 | 0.3* | 2002-2016 | -1.8* |  |  |  |  |  |  |  |  |
| Republic of Moldova | -0.9 | 1981-1985 | 4.8 | 1985-1992 | -5.9* | 1992-1995 | 6.1 | 1995-2004 | 1.8* | 2004-2016 | -3.4* |  |  |
| Russia | -1.5* | 1980-1987 | 1.6* | 1987-1991 | -2.5 | 1991-1994 | 4.2 | 1994-2004 | 0.9* | 2004-2015 | -6.7* |  |  |
| Ukraine | -0.8 | 1981-1985 | 5.9 | 1985-1991 | -1.7 | 1991-1994 | 4.5 | 1994-2015 | -2.6* |  |  |  |  |
| **Central Asia** | **-0.9** |  |  |  |  |  |  |  |  |  |  |  |  |
| Armenia | -1.6 | 1981-1993 | 2.5* | 1993-2000 | -4.7* | 2000-2003 | 17.5 | 2003-2016 | -7.4* |  |  |  |  |
| Azerbaijan | 0.8 | 1981-1987 | 1.6 | 1987-1990 | -13.6 | 1990-2001 | 0.7 | 2001-2007 | 8.2* |  |  |  |  |
| Georgia | -1.9 | 1981-2005 | -0.9* | 2005-2013 | -12.4* | 2013-2015 | 35.8 |  |  |  |  |  |  |
| Kazakhstan | -1.8* | 1981-2003 | 1.2* | 2003-2015 | -7.1* |  |  |  |  |  |  |  |  |
| Kyrgyzstan | -0.9 | 1981-1991 | 1.2* | 1991-1994 | 5.9 | 1994-1997 | -4.8 | 1997-1005 | 1.4 | 2005-2015 | -5.4* |  |  |
| Tajikistan | -0.8 | 1981-1994 | 0.3 | 1994-1998 | -20.8* | 1998-2004 | 12.7* |  |  |  |  |  |  |
| Turkmenistan | -6.3 | 1981-1987 | 0.7 | 1987-1990 | -22.3 | 1990-1994 | 20.2 | 1994-1998 | -24.5* |  |  |  |  |
| Uzbekistan | 0.9 | 1981-1995 | 2.7* | 1995-2005 | -1.6 |  |  |  |  |  |  |  |  |
| **Middle East and North Africa** |  |  |  |  |  |  |  |  |  |  |  |  |  |
| Turkey | -0.5 | 2009-2015 | -0.5 |  |  |  |  |  |  |  |  |  |  |

| **Colour** | **Average APC** | **Final segment only** |
| --- | --- | --- |
|  | Significant decrease | Significant decrease |
|  | - | Significant decrease but plateauing |
|  | No significant change | No significant change |
|  | Significant increase | Significant increase |
|  |  | No data available |

APC = Annual Percentage Change for one segment of a trend **Key:**

Average APC = Average APC for overall period

^a^AAPC for geographical regions = median values for constituent countries

*Rate of change significantly different from 0 at p<0.05
