# Supplementary material for: Epidemiology report: trends in sex-specific cerebrovascular disease mortality in Europe based on WHO mortality data
Source: Eur Heart J. 2018 Aug 14;40(9):755–64. doi: 10.1093/eurheartj/ehy378 (PMC6396027; doi:10.1093/eurheartj/ehy378)
Supplement: Supplementary Table S3 [file ehy378_supplementary_table_s3.docx]

**Supplementary Table 3: JoinPoint analysis of trends in age standardised mortality rates for ischaemic stroke by country, geographic region and sex**

**Males**

|  | **Total study period** | **Period 1** | | **Period 2** | | **Period 3** | | **Period 4** | | **Period 5** | | **Period 6** | |
| --- | --- | --- | --- | --- | --- | --- | --- | --- | --- | --- | --- | --- | --- |
|  | **Average APC (%)^a^** | **Years** | **APC (%)** | **Years** | **APC (%)** | **Years** | **APC (%)** | **Years** | **APC (%)** | **Years** | **APC (%)** | **Years** | **APC (%)** |
| **Western Europe** | **-3.9** |  |  |  |  |  |  |  |  |  |  |  |  |
| Austria | -3.2* | 1980-2002 | -1.1* | 2002-2005 | -14.5* | 2005-2016 | -4.2* |  |  |  |  |  |  |
| Belgium | -3.5* | 1980-1983 | -14.0* | 1983-1986 | 5.7 | 1986-1989 | -21.1 | 1989-2000 | 2.4* | 2000-2010 | -6.8* | 2010-2015 | 4.2 |
| Cyprus | -10.4 | 2004-2016 | -10.4 |  |  |  |  |  |  |  |  |  |  |
| Denmark | -1.9* | 1994-2009 | -0.2 | 2009-2015 | -6.0* |  |  |  |  |  |  |  |  |
| Finland | -3.9* | 1987-2001 | -2.9* | 2001-2008 | -7.6* | 2008-2014 | -2.0 |  |  |  |  |  |  |
| France | -2.3* | 1980-1997 | -11.3* | 1997-2001 | 48.1* | 2001-2014 | -2.5* |  |  |  |  |  |  |
| Germany | 1.7* | 1980-1982 | 14.9* | 1982-1997 | 1.5* | 1997-2003 | 3.5* | 2003-2015 | -0.9* |  |  |  |  |
| Greece | -6.9* | 1980-1992 | -10.7* | 1992-2002 | -21.9* | 2002-2005 | 33.7 | 2005-2011 | -12.7* | 2011-2015 | 37.4* |  |  |
| Iceland | -4.7* | 1981-1994 | -0.3 | 1994-2016 | -7.2* |  |  |  |  |  |  |  |  |
| Ireland | -8.0* | 1980-2003 | -8.5* | 2003-2011 | -0.7 | 2011-2013 | -27.7* |  |  |  |  |  |  |
| Israel | -1.4* | 1980-2015 | -1.4* |  |  |  |  |  |  |  |  |  |  |
| Italy | -7.5* | 1980-1983 | -5.5 | 1983-1993 | -15.8* | 1993-2001 | -3.2 | 2001-2005 | -17.8* | 2005-2015 | 2.0 |  |  |
| Luxembourg | -9.2* | 1980-2015 | -9.2* |  |  |  |  |  |  |  |  |  |  |
| Malta | -6.7* | 1980-2015 | -6.7* |  |  |  |  |  |  |  |  |  |  |
| Netherlands | -2.1* | 1980-1987 | -4.6* | 1987-1992 | 1.5 | 1992-2006 | -1.8* | 2006-2013 | -4.5* | 2013-2016 | 2.5 |  |  |
| Norway | -0.6 | 1986-2000 | 1.4* | 2000-2015 | -2.6* |  |  |  |  |  |  |  |  |
| Portugal | -10.4* | 1980-1988 | -16.1* | 1988-2014 | -8.6* |  |  |  |  |  |  |  |  |
| San Marino |  |  |  |  |  |  |  |  |  |  |  |  |  |
| Spain | -8.3* | 1980-1993 | -9.5* | 1993-2006 | -8.0* | 2006-2009 | -2.6 | 2009-2015 | -9.4* |  |  |  |  |
| Sweden | -3.7* | 1987-1997 | 1.1* | 1997-2003 | -3.3* | 2003-2006 | -12.3* | 2006-2009 | -0.3 | 2009-2015 | -8.9* |  |  |
| Switzerland | 1.7 | 1995-2000 | -15.2* | 2000-2003 | 47.2 | 2003-2013 | -0.3 |  |  |  |  |  |  |
| United K. | -6.1* | 1980-1998 | -8.4* | 1998-2002 | 5.5 | 2002-2015 | -6.3* |  |  |  |  |  |  |
| **Central Europe** | **2.4** |  |  |  |  |  |  |  |  |  |  |  |  |
| Albania | -2.2 | 1987-1994 | -9.8* | 1994-2000 | 8.6 | 2000-2010 | -2.7 |  |  |  |  |  |  |
| Bosnia | 9.1* | 1985-2014 | 9.1* |  |  |  |  |  |  |  |  |  |  |
| Bulgaria | 5.9* | 1980-1982 | 28.0 | 1982-1987 | -2.1 | 1987-1990 | 33.5 | 1990-2002 | -1.1 | 2002-2005 | 24.7 | 2005-2014 | 1.9 |
| Croatia | 2.6* | 1985-1995 | -7.6* | 1995-2016 | 7.8* |  |  |  |  |  |  |  |  |
| Czech R. | -2.7* | 1986-2002 | -4.9* | 2002-2012 | 2.2* | 2012-2016 | -5.7* |  |  |  |  |  |  |
| Hungary | -3.2* | 1980-2003 | -0.5* | 2003-2006 | -17.7* | 2006-2016 | -4.6* |  |  |  |  |  |  |
| Montenegro | 32.8* | 2000-2009 | 32.8* |  |  |  |  |  |  |  |  |  |  |
| Poland | 1.3* | 1980-1988 | 8.1* | 1988-1995 | -3.1* | 1995-2000 | 6.6* | 2000-2009 | 0.1 | 2009-2015 | -4.9* |  |  |
| Romania | -3.4* | 1980-1993 | -3.6* | 1993-1996 | -39.6* | 1996-1999 | 43.1* | 1999-2016 | -1.9* |  |  |  |  |
| Serbia | 3.2 | 1998-2003 | -1.6 | 2003-2006 | 24.7 | 2006-2015 | -0.5 |  |  |  |  |  |  |
| Slovakia | 2.9 | 1992-1995 | 13.1* | 1995-2006 | 2.2* | 2006-2009 | 13.9 | 2009-2014 | -7.1* |  |  |  |  |
| Slovenia | -5.6* | 1985-1992 | -5.3 | 1992-1998 | -26.6* | 1998-2015 | 3.1* |  |  |  |  |  |  |
| TFYR Macedonia | 2.4 | 1991-2013 | 2.4 |  |  |  |  |  |  |  |  |  |  |
| **Eastern Europe** | **-1.0** |  |  |  |  |  |  |  |  |  |  |  |  |
| Belarus |  |  |  |  |  |  |  |  |  |  |  |  |  |
| Estonia | -8.2* | 1994-2003 | -2.7* | 2003-2015 | -12.1* |  |  |  |  |  |  |  |  |
| Latvia | -1.8* | 1996-2005 | 1.6* | 2005-2015 | -4.7* |  |  |  |  |  |  |  |  |
| Lithuania | -0.2 | 1993-2016 | -0.2 |  |  |  |  |  |  |  |  |  |  |
| Republic of Moldova | 8.7* | 1991-2002 | 2.5 | 2002-2005 | 104.2* | 2005-2016 | -2.9 |  |  |  |  |  |  |
| Russia |  |  |  |  |  |  |  |  |  |  |  |  |  |
| Ukraine |  |  |  |  |  |  |  |  |  |  |  |  |  |
| **Central Asia** | **4.5** |  |  |  |  |  |  |  |  |  |  |  |  |
| Armenia | -5.7* | 2008-2016 | -5.7* |  |  |  |  |  |  |  |  |  |  |
| Azerbaijan |  |  |  |  |  |  |  |  |  |  |  |  |  |
| Georgia | 16.2 | 1998-2005 | -14.7 | 2005-2015 | 44.3* |  |  |  |  |  |  |  |  |
| Kazakhstan | 2.9* | 1991-2015 | 2.9* |  |  |  |  |  |  |  |  |  |  |
| Kyrgyzstan | 6.1 | 2000-2010 | -9.6* | 2010-2015 | 46.4* |  |  |  |  |  |  |  |  |
| Tajikistan |  |  |  |  |  |  |  |  |  |  |  |  |  |
| Turkmenistan |  |  |  |  |  |  |  |  |  |  |  |  |  |
| Uzbekistan |  |  |  |  |  |  |  |  |  |  |  |  |  |
| **North Africa and Middle East** |  |  |  |  |  |  |  |  |  |  |  |  |  |
| Turkey | 3.1 | 2009-2015 | 3.1 |  |  |  |  |  |  |  |  |  |  |

**Females**

|  | **Total study period** | **Period 1** | | **Period 2** | | **Period 3** | | **Period 4** | | **Period 5** | | **Period 6** | |
| --- | --- | --- | --- | --- | --- | --- | --- | --- | --- | --- | --- | --- | --- |
|  | **Average APC (%)^a^** | **Years** | **APC (%)** | **Years** | **APC (%)** | **Years** | **APC (%)** | **Years** | **APC (%)** | **Years** | **APC (%)** | **Years** | **APC (%)** |
| **Western Europe** | **-3.7** |  |  |  |  |  |  |  |  |  |  |  |  |
| Austria | -3.4* | 1980-1992 | -3.2* | 1992-2002 | -0.5 | 2002-2005 | -13.0* | 2005-2016 | -3.5* |  |  |  |  |
| Belgium | -3.7* | 1980-1983 | -14.5* | 1983-1986 | 6.3 | 1986-1989 | -20.6* | 1989-2003 | 0.4 | 2003-2007 | -12.4* | 2007-2015 | 1.7 |
| Cyprus | -9.6 | 2004-2016 | -9.6 |  |  |  |  |  |  |  |  |  |  |
| Denmark | -1.4* | 1994-2009 | 0.6 | 2009-2015 | -6.1* |  |  |  |  |  |  |  |  |
| Finland | -3.8* | 1987-2003 | -3.2* | 2003-2008 | -8.8* | 2008-2014 | -1.1 |  |  |  |  |  |  |
| France | -2.2* | 1980-1998 | -11.7* | 1998-2001 | 81.7* | 2001-2014 | -2.4* |  |  |  |  |  |  |
| Germany | 2.2* | 1980-1982 | 11.5 | 1982-1997 | 0.9* | 1997-2004 | 5.7* | 2004-2015 | 0.1 |  |  |  |  |
| Greece | -8.3* | 1980-1993 | -10.9* | 1993-2002 | -21.7* | 2002-2006 | 16.8 | 2006-2010 | -19.5* | 2010-2015 | 20.0* |  |  |
| Iceland | -3.6* | 1981- 1995 | 1.1 | 1995-2016 | -6.6* |  |  |  |  |  |  |  |  |
| Ireland | -7.8* | 1980-2003 | -9.4* | 2003-2013 | -3.9* |  |  |  |  |  |  |  |  |
| Israel | -2.6 | 1980-1990 | -12.3* | 1990-1993 | 48.9 | 1993-1996 | -24.1 | 1996-2015 | 0.1 |  |  |  |  |
| Italy | -7.8* | 1980-1983 | -5.4 | 1983-1994 | -15.8* | 1994-2001 | -2.7 | 2001-2004 | -21.9* | 2004-2015 | 1.5 |  |  |
| Luxembourg | -6.5 | 1980-2002 | -9.3* | 2002-2005 | -30.1 | 2005-2015 | 9.1* |  |  |  |  |  |  |
| Malta | -10.7* | 1980-1986 | -28.1* | 1986-1989 | 43.0 | 1989-2010 | -7.2* | 2010-2015 | -25.7* |  |  |  |  |
| Netherlands | -1.9* | 1980-1990 | -3.5* | 1990-1993 | 3.6 | 1993-1996 | -4.6 | 1996-2003 | 0.1 | 2003-2016 | -2.4* |  |  |
| Norway | -0.8 | 1986-1988 | -10.6 | 1988-2002 | 2.0* | 2002-2015 | -2.2* |  |  |  |  |  |  |
| Portugal | -10.7* | 1980-1988 | -16.5* | 1988-2014 | -8.8* |  |  |  |  |  |  |  |  |
| San Marino |  |  |  |  |  |  |  |  |  |  |  |  |  |
| Spain | -8.4* | 1980-1997 | -9.7* | 1997-2006 | -7.9* | 2006-2010 | -3.9 | 2010-2013 | -12.8* | 2013-2015 | -1.4 |  |  |
| Sweden | -2.7* | 1987-1999 | 1.4* | 1999-2015 | -5.7* |  |  |  |  |  |  |  |  |
| Switzerland | 2.7 | 1995-2000 | -13.5* | 2000-2003 | 46.2 | 2003-2013 | 0.7 |  |  |  |  |  |  |
| United K. | -6.1* | 1980-1998 | -9.2* | 1998-2002 | 5.8 | 2002-2015 | -5.3* |  |  |  |  |  |  |
| **Central Europe** | **2.1** |  |  |  |  |  |  |  |  |  |  |  |  |
| Albania | -0.9 | 1987-2010 | -0.9 |  |  |  |  |  |  |  |  |  |  |
| Bosnia | 8.8* | 1985-2014 | 8.8* |  |  |  |  |  |  |  |  |  |  |
| Bulgaria | 5.6* | 1980-1982 | 28.7 | 1982-1987 | -2.8 | 1987-1990 | 31.3 | 1990-2002 | -1.1 | 2002-2005 | 24.3 | 2005-2014 | 1.6 |
| Croatia | 2.1 | 1985-1995 | -9.8* | 1995-1998 | 29.6 | 1998-2001 | -19.8 | 2001-2007 | 23.8* | 2007-2016 | 3.2 |  |  |
| Czech R. | -2.3* | 1986-1992 | -7.4* | 1992-1995 | 1.2 | 1995-2004 | -5.3* | 2004-2009 | 8.5* | 2009-2016 | -2.6* |  |  |
| Hungary | -3.4* | 1980-2003 | -0.9* | 2003-2006 | -18.9* | 2006-2016 | -4.1* |  |  |  |  |  |  |
| Montenegro | 34.1* | 2000-2009 | 34.1* |  |  |  |  |  |  |  |  |  |  |
| Poland | 1.1* | 1980-1986 | 11.0* | 1986-1995 | -2.3* | 1995-2000 | 5.3 | 2000-2010 | -0.3 | 2010-2015 | -4.7* |  |  |
| Romania | -3.7* | 1980-1992 | -2.3* | 1992-1995 | -41.1 | 1995-1999 | 22.7* | 1999-2016 | -1.8* |  |  |  |  |
| Serbia | 3.8 | 1998-2003 | -0.7 | 2003-2006 | 28.1* | 2006-2015 | -0.7 |  |  |  |  |  |  |
| Slovakia | 3.0* | 1992-1994 | 19.0 | 1994-2006 | 1.7* | 2006-2009 | 16.7 | 2009-2014 | -7.2* |  |  |  |  |
| Slovenia | -5.5* | 1985-1993 | -9.1* | 1993-1998 | -29.6* | 1998-2015 | 4.9* |  |  |  |  |  |  |
| TFYR Macedonia | 3.4* | 1991-2013 | 3.4* |  |  |  |  |  |  |  |  |  |  |
| **Eastern Europe** | **-0.9** |  |  |  |  |  |  |  |  |  |  |  |  |
| Belarus |  |  |  |  |  |  |  |  |  |  |  |  |  |
| Estonia | -9.0* | 1994-2003 | -3.0* | 2003-2015 | -13.2* |  |  |  |  |  |  |  |  |
| Latvia | -1.3* | 1996-2003 | 2.8* | 2003-2015 | -3.6* |  |  |  |  |  |  |  |  |
| Lithuania | -0.4 | 1993-2016 | -0.4 |  |  |  |  |  |  |  |  |  |  |
| Republic of Moldova | 8.6* | 1991-2002 | 2.3* | 2002-2005 | 103.7* | 2005-2016 | -3.0* |  |  |  |  |  |  |
| Russia |  |  |  |  |  |  |  |  |  |  |  |  |  |
| Ukraine |  |  |  |  |  |  |  |  |  |  |  |  |  |
| **Central Asia** | **3.8** |  |  |  |  |  |  |  |  |  |  |  |  |
| Armenia | -4.0* | 2008-2016 | -4.0* |  |  |  |  |  |  |  |  |  |  |
| Azerbaijan |  |  |  |  |  |  |  |  |  |  |  |  |  |
| Georgia | 13.5 | 1998-2005 | -22.5 | 2005-2015 | 48.3* |  |  |  |  |  |  |  |  |
| Kazakhstan | 2.8* | 1991-2015 | 2.8* |  |  |  |  |  |  |  |  |  |  |
| Kyrgyzstan | 4.8 | 2000-2010 | -10.5* | 2010-2015 | 43.8* |  |  |  |  |  |  |  |  |
| Tajikistan |  |  |  |  |  |  |  |  |  |  |  |  |  |
| Turkmenistan |  |  |  |  |  |  |  |  |  |  |  |  |  |
| Uzbekistan |  |  |  |  |  |  |  |  |  |  |  |  |  |
| **North Africa and Middle East** |  |  |  |  |  |  |  |  |  |  |  |  |  |
| Turkey | 2.8 | 2009-2015 | 2.8 |  |  |  |  |  |  |  |  |  |  |

| **Colour** | **Average APC** | **Final segment only** |
| --- | --- | --- |
|  | Significant decrease | Significant decrease |
|  | - | Significant decrease but plateauing |
|  | No significant change | No significant change |
|  | Significant increase | Significant increase |
|  | - | No data available |

APC = Annual Percentage Change for one segment of a trend **Key:**

Average APC = Average APC for overall period

^a^AAPC for geographical regions = median values for constituent countries

*Rate of change significantly different from 0 at p<0.05
