# Supplementary material for: Epidemiology report: trends in sex-specific cerebrovascular disease mortality in Europe based on WHO mortality data
Source: Eur Heart J. 2018 Aug 14;40(9):755–64. doi: 10.1093/eurheartj/ehy378 (PMC6396027; doi:10.1093/eurheartj/ehy378)
Supplement: Supplementary Table S4 [file ehy378_supplementary_table_s4.docx]

**Supplementary Table 4: JoinPoint analysis of trends in age standardised mortality rates for haemorrhagic stroke by country, geographic region and sex**

**Males**

|  | **Total study period** | **Period 1** | | **Period 2** | | **Period 3** | | **Period 4** | | **Period 5** | | **Period 6** | |
| --- | --- | --- | --- | --- | --- | --- | --- | --- | --- | --- | --- | --- | --- |
|  | **Average APC (%)^a^** | **Years** | **APC (%)** | **Years** | **APC (%)** | **Years** | **APC (%)** | **Years** | **APC (%)** | **Years** | **APC (%)** | **Years** | **APC (%)** |
| **Western Europe** | **-1.5** |  |  |  |  |  |  |  |  |  |  |  |  |
| Austria | -1.3 | 1980-2001 | 0.6* | 2001-2004 | -16.1 | 2004-2016 | -0.5 |  |  |  |  |  |  |
| Belgium | -0.6* | 1980-1987 | -3.4* | 1987-1996 | 5.3* | 1996-2015 | -2.2* |  |  |  |  |  |  |
| Cyprus | 0.5 | 2004-2016 | 0.5 |  |  |  |  |  |  |  |  |  |  |
| Denmark | -2.5* | 1994-2004 | -0.5 | 2004-2015 | -4.3* |  |  |  |  |  |  |  |  |
| Finland | -0.9* | 1987-1995 | 2.3* | 1995-2009 | -3.3* | 2009-2014 | 0.6 |  |  |  |  |  |  |
| France | -2.5* | 1980-1990 | -4.5* | 1990-1998 | 0.4 | 1998-2014 | -2.6* |  |  |  |  |  |  |
| Germany | -1.3* | 1980-1989 | -3.1* | 1998-1996 | 2.9* | 1996-2015 | -2.0* |  |  |  |  |  |  |
| Greece | -1.9 | 1980-1989 | -7.8* | 1989-1992 | 6.4 | 1992-1995 | -8.6 | 1995-2005 | 0.5 | 2005-2010 | 3.8* | 2010-2015 | 6.0* |
| Iceland | -3.6* | 1981-2016 | -3.6* |  |  |  |  |  |  |  |  |  |  |
| Ireland | -4.4* | 1980-1995 | -7.7* | 1995-2013 | 0.8 |  |  |  |  |  |  |  |  |
| Israel | 0.5 | 1980-1988 | -3.0 | 1988-1996 | 9.8* | 1996-2010 | -3.8* | 2010-2015 | 3.8 |  |  |  |  |
| Italy | -1.5* | 1980-1991 | -5.6* | 1991-2000 | 3.6* | 2000-2015 | -1.4* |  |  |  |  |  |  |
| Luxembourg | -2.7* | 1980-1993 | -4.7* | 1993-2000 | 8.1 | 2000-2015 | -5.7* |  |  |  |  |  |  |
| Malta | -4.1 | 1980-1984 | -21.1* | 1984-1987 | 105.6* | 1987-1990 | -53.0* | 1990-2015 | -1.7* |  |  |  |  |
| Netherlands | -1.2 | 1980-1990 | -0.1 | 1990-1993 | 7.6 | 1993-1999 | -3.8* | 1999-2004 | 2.2 | 2004-2016 | -4.2* |  |  |
| Norway | -0.1 | 1986-1988 | -8.7 | 1988-1998 | 5.0* | 1998-2015 | -2.0* |  |  |  |  |  |  |
| Portugal | -0.5 | 1980-1982 | -11.2 | 1982-1990 | -0.2 | 19990-1999 | 5.7* | 1999-2014 | -2.8* |  |  |  |  |
| San Marino |  |  |  |  |  |  |  |  |  |  |  |  |  |
| Spain | -1.9* | 1980-1998 | -4.0* | 1989-2005 | 0.2 | 2005-2015 | -3.4* |  |  |  |  |  |  |
| Sweden | -1.6* | 1987-1998 | 0.5 | 1998-2009 | -4.2* | 2009-2015 | -0.6 |  |  |  |  |  |  |
| Switzerland | -1.5 | 1995-1998 | 10.0* | 1998-2013 | -3.7* |  |  |  |  |  |  |  |  |
| United K. | -2.0* | 1980-1989 | -9.9* | 1989-1994 | -0.2 | 1994-2002 | 1.9* | 2002-2006 | -1.7 | 2006-2013 | 0.6 | 2013-2015 | 5.3 |
| **Central Europe** | **-3.2** |  |  |  |  |  |  |  |  |  |  |  |  |
| Albania | -0.6 | 1987-2000 | 3.2* | 2000-2010 | -5.3* |  |  |  |  |  |  |  |  |
| Bosnia | -0.0 | 1985-2014 | -0.0 |  |  |  |  |  |  |  |  |  |  |
| Bulgaria | -2.9* | 1980-2003 | 1.1* | 2003-2006 | -22.6 | 2006-2014 | -5.7* |  |  |  |  |  |  |
| Croatia | -0.6 | 1985-1998 | 3.2* | 1998-2001 | -8.9 | 2001-2016 | -2.1* |  |  |  |  |  |  |
| Czech republic | -3.3* | 1986-1998 | -7.2* | 1998-2016 | -0.6 |  |  |  |  |  |  |  |  |
| Hungary | -4.9* | 1980-1988 | -6.1* | 1988-1999 | -3.8* | 1999-2007 | -8.0* | 2007-2013 | -4.4* | 2013-2016 | 1.9 |  |  |
| Montenegro | -7.4* | 2000-2005 | 2.1 | 2005-2009 | -18.1* |  |  |  |  |  |  |  |  |
| Poland | -3.4* | 1980-1983 | -9.9* | 1983-2003 | -3.1* | 2003-2009 | -0.7 | 2009-2015 | -3.6* |  |  |  |  |
| Romania | -3.8* | 1980-1991 | -6.7* | 1991-1994 | 13.5 | 1994-2003 | -3.4* | 2003-2008 | -8.2* | 2008-2016 | -3.5* |  |  |
| Serbia | -3.2* | 1998-2005 | 2.6* | 2005-2015 | -3.6* |  |  |  |  |  |  |  |  |
| Slovakia | -3.2* | 1992-2003 | -8.8* | 2003-2009 | 8.9* | 2009-2014 | -4.2 |  |  |  |  |  |  |
| Slovenia | -1.6* | 1985-2002 | 1.0 | 2002-2015 | -5.0* |  |  |  |  |  |  |  |  |
| TFYR Macedonia | 1.8 | 1991-1998 | 6.0 | 1998-2002 | -10.1 | 2002-2013 | 3.7* |  |  |  |  |  |  |
| **Eastern Europe** | **-2.9** |  |  |  |  |  |  |  |  |  |  |  |  |
| Belarus |  |  |  |  |  |  |  |  |  |  |  |  |  |
| Estonia | -7.2* | 1994-2015 | -7.2* |  |  |  |  |  |  |  |  |  |  |
| Latvia | -5.3* | 1996-2015 | -5.3* |  |  |  |  |  |  |  |  |  |  |
| Lithuania | -0.4 | 1993-2016 | -0.4 |  |  |  |  |  |  |  |  |  |  |
| Republic of Moldova | 0.0 | 1991-2002 | 8.5* | 2002-2016 | -6.2* |  |  |  |  |  |  |  |  |
| Russia |  |  |  |  |  |  |  |  |  |  |  |  |  |
| Ukraine |  |  |  |  |  |  |  |  |  |  |  |  |  |
| **Central Asia** | **-4.7** |  |  |  |  |  |  |  |  |  |  |  |  |
| Armenia | -9.6* | 2008-2016 | -9.6* |  |  |  |  |  |  |  |  |  |  |
| Azerbaijan |  |  |  |  |  |  |  |  |  |  |  |  |  |
| Georgia | -3.0 | 1998-2000 | -21.6 | 2000-2005 | 29.7 | 2005-2009 | -2.9 | 2009-2012 | -47.7 | 2012-2015 | 27.5 |  |  |
| Kazakhstan | -3.2* | 1991-1997 | 1.1 | 1997-2000 | -28.5* | 2000-2015 | 1.0 |  |  |  |  |  |  |
| Kyrgyzstan | -6.1 | 2000-2006 | -20.9* | 2006-2009 | 41.4 | 2009-2012 | -42.8 | 2012-2015 | 44.5* |  |  |  |  |
| Tajikistan |  |  |  |  |  |  |  |  |  |  |  |  |  |
| Turkmenistan |  |  |  |  |  |  |  |  |  |  |  |  |  |
| Uzbekistan |  |  |  |  |  |  |  |  |  |  |  |  |  |
| **Middle East and North Africa** |  |  |  |  |  |  |  |  |  |  |  |  |  |
| Turkey | -1.6 | 2009-2015 | -1.6 |  |  |  |  |  |  |  |  |  |  |

**Females**

|  | **Total study period** | **Period 1** | | **Period 2** | | **Period 3** | | **Period 4** | | **Period 5** | | **Period 6** | |
| --- | --- | --- | --- | --- | --- | --- | --- | --- | --- | --- | --- | --- | --- |
|  | **Average APC (%)^a^** | **Years** | **APC (%)** | **Years** | **APC (%)** | **Years** | **APC (%)** |  |  | **Years** | **APC (%)** | **Years** | **APC (%)** |
| **Western Europe** | **-2.2** |  |  |  |  |  |  |  |  |  |  |  |  |
| Austria | -1.2* | 1980-1989 | -1.5* | 1989-1999 | 2.7* | 1999-2006 | -7.7* | 2006-2016 | 0.1 |  |  |  |  |
| Belgium | -0.9* | 1980-1988 | -2.9* | 1988-1996 | 3.7* | 1996-2015 | -2.0* |  |  |  |  |  |  |
| Cyprus | -2.6 | 2004-2016 | -2.6 |  |  |  |  |  |  |  |  |  |  |
| Denmark | -2.2* | 1994-2005 | -1.5* | 2005-2013 | -5.1* | 2013-2015 | 5.8 |  |  |  |  |  |  |
| Finland | -1.8* | 1987-1995 | 0.2 | 1995-2005 | -4.1* | 2005-2014 | -0.9* |  |  |  |  |  |  |
| France | -2.5* | 1980-1989 | -5.6* | 1989-1995 | -0.5 | 1995-1999 | 2.5 | 1999-2002 | -4.6 | 2002-2009 | -1.2* | 2009-2014 | -3.7* |
| Germany | -1.1* | 1980-1982 | 1.8 | 1982-1989 | -4.1* | 1989-1996 | 1.4* | 1996-2008 | -1.2* | 2008-2011 | -3.8 | 2011-2015 | 0.5 |
| Greece | -3.7* | 1980-1989 | -9.5* | 1989-1992 | 1.9 | 1992-1995 | -12.6 | 1995-2015 | -0.3 |  |  |  |  |
| Iceland | -3.6* | 1981-2016 | -3.6* |  |  |  |  |  |  |  |  |  |  |
| Ireland | -4.4* | 1980-1992 | -9.3* | 1992-2005 | -2.8* | 2005-2009 | 10.6* | 2009-2013 | -8.5* |  |  |  |  |
| Israel | -1.3 | 1980-1990 | -3.8* | 1990-1993 | 14.9 | 1993-2015 | -2.2* |  |  |  |  |  |  |
| Italy | -2.1* | 1980-1990 | -7.2* | 1990-1999 | 2.3* | 1999-2015 | -1.2* |  |  |  |  |  |  |
| Luxembourg | -4.3* | 1980-1983 | -19.6 | 1983-2002 | 1.6 | 2002-2015 | -8.8* |  |  |  |  |  |  |
| Malta | -6.8 | 1980-1984 | -34.8* | 1984-1987 | 120.2* | 1987-1990 | -52.3 | 1990-2015 | -3.5* |  |  |  |  |
| Netherlands | -1.3 | 1980-1989 | -2.8* | 1989-1992 | 6.5 | 1992-2001 | -2.3* | 2001-2004 | 4.4 | 2004-2016 | -2.7* |  |  |
| Norway | -0.4 | 1986-1998 | -8.6 | 1998-1997 | 4.3* | 1997-2015 | -1.7* |  |  |  |  |  |  |
| Portugal | -1.2 | 1980-1986 | -6.6* | 1986-1993 | 0.9 | 1993-1997 | 9.9* | 1997-2014 | -2.6* |  |  |  |  |
| San Marino |  |  |  |  |  |  |  |  |  |  |  |  |  |
| Spain | -2.7* | 1980-1989 | -5.5* | 1989-1995 | -2.5* | 1995-2000 | 0.6 | 2000-2008 | -1.1* | 2008-2012 | -4.9* | 2012-2015 | -1.1 |
| Sweden | -2.3* | 1987-1995 | -2.4* | 1995-1999 | 1.1 | 1999-2009 | -4.2* | 2009-2015 | -1.0 |  |  |  |  |
| Switzerland | -2.1* | 1995-2013 | -2.1* |  |  |  |  |  |  |  |  |  |  |
| United K. | -2.3* | 1980-1988 | -9.5* | 1988-1992 | -4.9* | 1992-2008 | 0.3* | 2008-2015 | 1.9* |  |  |  |  |
| **Central Europe** | **-3.7** |  |  |  |  |  |  |  |  |  |  |  |  |
| Albania | 0.4 | 1987-2000 | 3.0* | 2000-2010 | -2.9 |  |  |  |  |  |  |  |  |
| Bosnia | -0.5 | 1985-1988 | 8.5 | 1988-2014 | -1.5* |  |  |  |  |  |  |  |  |
| Bulgaria | -3.7* | 1980-2003 | 0.5 | 2003-2006 | -24.2* | 2006-2014 | -6.9* |  |  |  |  |  |  |
| Croatia | -1.1 | 1985-1998 | 2.7* | 1998-2001 | -10.9 | 2001-2016 | -2.3* |  |  |  |  |  |  |
| Czech R. | -3.7* | 1986-1993 | -7.5* | 1993-1997 | -12.2* | 1997-2016 | -0.3 |  |  |  |  |  |  |
| Hungary | -6.1* | 1980-2000 | -5.9* | 2000-2007 | -10.0* | 2007-2016 | -3.3* |  |  |  |  |  |  |
| Montenegro | -5.6* | 2000-2004 | 4.5 | 2004-2009 | -13.0* |  |  |  |  |  |  |  |  |
| Poland | -4.6* | 1980-1983 | -11.2* | 1983-1999 | -4.0* | 1999-2003 | -6.4* | 2003-2015 | -2.9* |  |  |  |  |
| Romania | -4.8* | 1980-1991 | -7.3* | 1991-1994 | 11.1 | 1994-2002 | -3.9* | 2002-2016 | -6.4* |  |  |  |  |
| Serbia | -3.2* | 1998-2006 | 0.6 | 2006-2015 | -6.5* |  |  |  |  |  |  |  |  |
| Slovakia | -4.6* | 1992-2000 | -12.8* | 2000-2005 | -4.0 | 2005-2008 | 16.4 | 2008-2014 | -3.2 |  |  |  |  |
| Slovenia | -1.2* | 1985-2015 | -1.2* |  |  |  |  |  |  |  |  |  |  |
| TFYR Macedonia | 0.3 | 1991-2013 | 0.3 |  |  |  |  |  |  |  |  |  |  |
| **Eastern Europe** | **-4.3** |  |  |  |  |  |  |  |  |  |  |  |  |
| Belarus |  |  |  |  |  |  |  |  |  |  |  |  |  |
| Estonia | -8.6* | 1994-2015 | -8.6* |  |  |  |  |  |  |  |  |  |  |
| Latvia | -6.1* | 1996-2004 | -4.2* | 2004-2009 | -12.2* | 2009-2015 | -3.3* |  |  |  |  |  |  |
| Lithuania | -2.5* | 1993-2016 | -2.5* |  |  |  |  |  |  |  |  |  |  |
| Republic of Moldova | -0.6 | 1991-2002 | 8.9* | 2002-2016 | -7.5* |  |  |  |  |  |  |  |  |
| Russia |  |  |  |  |  |  |  |  |  |  |  |  |  |
| Ukraine |  |  |  |  |  |  |  |  |  |  |  |  |  |
| **Central Asia** | **-4.3** |  |  |  |  |  |  |  |  |  |  |  |  |
| Armenia | -12.0* | 2008-2016 | -12.0* |  |  |  |  |  |  |  |  |  |  |
| Azerbaijan |  |  |  |  |  |  |  |  |  |  |  |  |  |
| Georgia | -2.5 | 1998-2009 | 10.7* | 2009-2012 | -52.1 | 2012-2015 | 24.7 |  |  |  |  |  |  |
| Kazakhstan | -4.1* | 1991-1997 | 1.0 | 1997-2000 | -27.6 | 2000-2015 | -0.7 |  |  |  |  |  |  |
| Kyrgyzstan | -4.5 | 2000-2013 | -11.9* | 2013-2015 | 61.5 |  |  |  |  |  |  |  |  |
| Tajikistan |  |  |  |  |  |  |  |  |  |  |  |  |  |
| Turkmenistan |  |  |  |  |  |  |  |  |  |  |  |  |  |
| Uzbekistan |  |  |  |  |  |  |  |  |  |  |  |  |  |
| **Middle East and North Africa** |  |  |  |  |  |  |  |  |  |  |  |  |  |
| Turkey | -4.1 | 2009-2015 | -4.1 |  |  |  |  |  |  |  |  |  |  |

| **Colour** | **Average APC** | **Final segment only** |
| --- | --- | --- |
|  | Significant decrease | Significant decrease |
|  | - | Significant decrease but plateauing |
|  | No significant change | No significant change |
|  | Significant increase | Significant increase |
|  | - | No data available |

APC = Annual Percentage Change for one segment of a trend **Key:**

Average APC = Average APC for overall period

^a^AAPC for geographical regions = median values for constituent countries

*Rate of change significantly different from 0 at p<0.05
