# Supplementary material for: Epidemiology report: trends in sex-specific cerebrovascular disease mortality in Europe based on WHO mortality data
Source: Eur Heart J. 2018 Aug 14;40(9):755–64. doi: 10.1093/eurheartj/ehy378 (PMC6396027; doi:10.1093/eurheartj/ehy378)
Supplement: Supplementary Table S5 [file ehy378_supplementary_table_s5.docx]

**Supplementary Table 5: Joinpoint analysis of trends in age standardised mortality rates for subarachnoid haemorrhage by country, geographic region and sex**

**Males**

|  | **Total study period** | **Period 1** | | **Period 2** | | **Period 3** | | **Period 4** | | **Period 5** | | **Period 6** | |
| --- | --- | --- | --- | --- | --- | --- | --- | --- | --- | --- | --- | --- | --- |
|  | **Average APC (%)^a^** | **Years** | **APC (%)** | **Years** | **APC (%)** | **Years** | **APC (%)** | **Years** | **APC (%)** | **Years** | **APC (%)** | **Years** | **APC (%)** |
| **Western Europe** | **-2.2** |  |  |  |  |  |  |  |  |  |  |  |  |
| Austria | -0.2 | 1980-2000 | 1.0 | 2000-2003 | -11.3 | 2003-2016 | 0.8 |  |  |  |  |  |  |
| Belgium | 0.0 | 1980-1992 | -6.2* | 1992-1995 | 48.7 | 1995-2015 | -2.1* |  |  |  |  |  |  |
| Cyprus | -4.8 | 2004-2016 | -4.8 |  |  |  |  |  |  |  |  |  |  |
| Denmark | -1.9* | 1994-2015 | -1.9* |  |  |  |  |  |  |  |  |  |  |
| Finland | -3.8* | 1987-2014 | -3.8* |  |  |  |  |  |  |  |  |  |  |
| France | -3.3* | 1980-1992 | -8.9* | 1992-1995 | 7.8 | 1995-2014 | -1.3* |  |  |  |  |  |  |
| Germany | -1.4* | 1980-1984 | -4.2 | 1984-1999 | 2.4* | 1999-2015 | -4.2* |  |  |  |  |  |  |
| Greece | 0.2 | 1980-1986 | 1.0 | 1986-1989 | -21.5 | 1989-2003 | 3.3* | 2003-2008 | -34.3* | 2008-2015 | 40.7* |  |  |
| Iceland | -2.6* | 1981-2016 | -2.6* |  |  |  |  |  |  |  |  |  |  |
| Ireland | -2.3* | 1980-1994 | -5.7* | 1994-2013 | 0.2 |  |  |  |  |  |  |  |  |
| Israel | -5.6* | 1980-1986 | -21.8* | 1986-2015 | -1.9* |  |  |  |  |  |  |  |  |
| Italy | -3.0* | 1980-1990 | -8.1* | 1990-2001 | 1.9* | 2001-2004 | -8.5 | 2004-2015 | -1.6* |  |  |  |  |
| Luxembourg | 0.2 | 1980-2015 | 0.2 |  |  |  |  |  |  |  |  |  |  |
| Malta | -2.2 | 1980-2015 | -2.2 |  |  |  |  |  |  |  |  |  |  |
| Netherlands | -3.7* | 1980-1982 | -16.1 | 1982-2012 | -2.1* | 2012-2016 | -8.6* |  |  |  |  |  |  |
| Norway | -2.0* | 1986-2015 | -2.0* |  |  |  |  |  |  |  |  |  |  |
| Portugal | -1.3 | 1980-1983 | 23.1* | 1983-1987 | -17.8* | 1987-1991 | 4.9 | 1991-1994 | -16.0 | 1994-2014 | 0.2 |  |  |
| San Marino |  |  |  |  |  |  |  |  |  |  |  |  |  |
| Spain | -0.6* | 1980-1987 | -4.5* | 1987-1997 | 3.1* | 1997-2015 | -1.2* |  |  |  |  |  |  |
| Sweden | -2.7* | 1987-2015 | -2.7* |  |  |  |  |  |  |  |  |  |  |
| Switzerland | -0.2 | 1995-2013 | -0.2 |  |  |  |  |  |  |  |  |  |  |
| United K. | -2.7* | 1980-1989 | -4.4* | 1989-2001 | -0.1 | 2001-2009 | -5.2* | 2009-2015 | -1.7 |  |  |  |  |
| **Central Europe** | **-1.3** |  |  |  |  |  |  |  |  |  |  |  |  |
| Albania | 1.8 | 1987-1999 | -8.2 | 1999-2002 | 81.1 | 2002-2010 | -4.1 |  |  |  |  |  |  |
| Bosnia | -1.3* | 1985-2014 | -1.3* |  |  |  |  |  |  |  |  |  |  |
| Bulgaria | 0.6 | 1980-1986 | -8.2* | 1986-1993 | 10.9* | 1993-2002 | -4.0 | 2002-2014 | 2.9* |  |  |  |  |
| Croatia | -0.9* | 1985-2016 | -0.9* |  |  |  |  |  |  |  |  |  |  |
| Czech R. | -1.8* | 1986-1999 | -4.5* | 1999-2016 | 0.3 |  |  |  |  |  |  |  |  |
| Hungary | -2.0* | 1980-2000 | -1.1* | 2000-2016 | -3.1* |  |  |  |  |  |  |  |  |
| Montenegro | -20.5 | 2000-2009 | -20.5 |  |  |  |  |  |  |  |  |  |  |
| Poland | -1.3* | 1980-1988 | -3.0* | 1988-2015 | -2.5* |  |  |  |  |  |  |  |  |
| Romania | 4.1* | 1980-2016 | 4.1* |  |  |  |  |  |  |  |  |  |  |
| Serbia | -3.2* | 1998-2001 | 1.3 | 2001-2009 | -5.6* | 2009-2013 | 2.8 | 2013-2015 | -11.7 |  |  |  |  |
| Slovakia | -3.4* | 1992-2014 | -3.4* |  |  |  |  |  |  |  |  |  |  |
| Slovenia | 0.0 | 1985-2015 | 0.0 |  |  |  |  |  |  |  |  |  |  |
| TFYR Macedonia | 1.6 | 1991-2013 | 1.6 |  |  |  |  |  |  |  |  |  |  |
| **Eastern Europe** | **-2.9** |  |  |  |  |  |  |  |  |  |  |  |  |
| Belarus |  |  |  |  |  |  |  |  |  |  |  |  |  |
| Estonia | -3.7* | 1994-2015 | -3.7* |  |  |  |  |  |  |  |  |  |  |
| Latvia | -3.4* | 1996-2015 | -3.4* |  |  |  |  |  |  |  |  |  |  |
| Lithuania | -2.3* | 1993-2016 | -2.3* |  |  |  |  |  |  |  |  |  |  |
| Republic of Moldova | 1.7 | 1991-1998 | 15.7* | 1998-2016 | -3.3* |  |  |  |  |  |  |  |  |
| Russia |  |  |  |  |  |  |  |  |  |  |  |  |  |
| Ukraine |  |  |  |  |  |  |  |  |  |  |  |  |  |
| **Central Asia** | **-2.1** |  |  |  |  |  |  |  |  |  |  |  |  |
| Armenia | -9.4* | 2008-2014 | -16.2 | 2014-2016 | 14.6 |  |  |  |  |  |  |  |  |
| Azerbaijan |  |  |  |  |  |  |  |  |  |  |  |  |  |
| Georgia | 1.1 | 1998-2004 | -45.5 | 2004-2015 | 41.7* |  |  |  |  |  |  |  |  |
| Kazakhstan | -2.4 | 1991-1998 | 15.1* | 1998-2001 | -47.6 | 2001-2015 | 2.7 |  |  |  |  |  |  |
| Kyrgyzstan | -1.7 | 2000-2015 | -1.7 |  |  |  |  |  |  |  |  |  |  |
| Tajikistan |  |  |  |  |  |  |  |  |  |  |  |  |  |
| Turkmenistan |  |  |  |  |  |  |  |  |  |  |  |  |  |
| Uzbekistan |  |  |  |  |  |  |  |  |  |  |  |  |  |
| **Middle East and North Africa** |  |  |  |  |  |  |  |  |  |  |  |  |  |
| Turkey | 3.1* | 2009-2015 | 3.1* |  |  |  |  |  |  |  |  |  |  |

**Females**

|  | **Total study period** | **Period 1** | | **Period 2** | | **Period 3** | | **Period 4** | | **Period 5** | | **Period 6** | |
| --- | --- | --- | --- | --- | --- | --- | --- | --- | --- | --- | --- | --- | --- |
|  | **Average APC (%)^a^** | **Years** | **APC (%)** | **Years** | **APC (%)** | **Years** | **APC (%)** | **Years** | **APC (%)** | **Years** | **APC (%)** | **Years** | **APC (%)** |
| **Western Europe** | **-1.4** |  |  |  |  |  |  |  |  |  |  |  |  |
| Austria | 0.3 | 1980-2001 | 1.3* | 2001-2004 | -10.5 | 2004-2016 | 1.3 |  |  |  |  |  |  |
| Belgium | 0.6 | 1980-1990 | -6.3* | 1990-1997 | 18.9* | 1997-2015 | -2.0* |  |  |  |  |  |  |
| Cyprus | 3.9 | 2004-2016 | 3.9 |  |  |  |  |  |  |  |  |  |  |
| Denmark | -2.4* | 1994-2015 | -2.4* |  |  |  |  |  |  |  |  |  |  |
| Finland | -3.3* | 1987-2014 | -3.3* |  |  |  |  |  |  |  |  |  |  |
| France | -2.2* | 1980-1990 | -8.4* | 1990-2014 | 0.5* |  |  |  |  |  |  |  |  |
| Germany | -0.4 | 1980-1986 | -1.4 | 1986-1999 | 3.3* | 1999-2015 | -3.0* |  |  |  |  |  |  |
| Greece | 0.9 | 1980-2004 | -1.1 | 2004-2008 | -36.9* | 2008-2015 | 41.2* |  |  |  |  |  |  |
| Iceland | -4.1* | 1981-2015 | -4.1* |  |  |  |  |  |  |  |  |  |  |
| Ireland | -2.9* | 1980-1990 | -5.9* | 1990-2013 | -1.5* |  |  |  |  |  |  |  |  |
| Israel | -5.2* | 1980-1984 | -20.9* | 1984-2015 | -2.9* |  |  |  |  |  |  |  |  |
| Italy | -2.1* | 1980-1992 | -4.5* | 1992-1997 | 6.0* | 1997-2015 | -2.6* |  |  |  |  |  |  |
| Luxembourg | 2.6* | 1980-2015 | 2.6* |  |  |  |  |  |  |  |  |  |  |
| Malta | 0.2 | 1980-2015 | 0.2 |  |  |  |  |  |  |  |  |  |  |
| Netherlands | -1.4* | 1980-2001 | -0.4 | 2001-2016 | -2.9* |  |  |  |  |  |  |  |  |
| Norway | -2.3* | 1986-2015 | -2.3* |  |  |  |  |  |  |  |  |  |  |
| Portugal | -1.1 | 1980-1984 | 7.1 | 1984-1987 | -16.4 | 1987-1991 | 4.1 | 1991-1994 | -11.1 | 1994-2007 | 2.0 | 2007-2014 | -2.6 |
| San Marino |  |  |  |  |  |  |  |  |  |  |  |  |  |
| Spain | 0.3 | 1980-1987 | -1.4 | 1987-2001 | 3.6* | 2001-2015 | -2.0* |  |  |  |  |  |  |
| Sweden | -2.7* | 1987-2003 | -1.3* | 2003-2015 | -4.4* |  |  |  |  |  |  |  |  |
| Switzerland | -0.2 | 1995-2013 | 0.2 |  |  |  |  |  |  |  |  |  |  |
| United K. | -2.5* | 1980-1984 | -2.2* | 1984-1990 | -4.5* | 1990-2001 | -0.3 | 2001-2010 | -5.2* | 2010-2015 | -0.3 |  |  |
| **Central Europe** | **-1.2** |  |  |  |  |  |  |  |  |  |  |  |  |
| Albania | -4.9 | 1987-1997 | -12.8 | 1997-2005 | 37.2* | 2005-2010 | -37.0 |  |  |  |  |  |  |
| Bosnia | -1.2 | 1985-2014 | -1.2 |  |  |  |  |  |  |  |  |  |  |
| Bulgaria | 1.6* | 1980-2014 | 1.6* |  |  |  |  |  |  |  |  |  |  |
| Croatia | -0.9 | 1985-1989 | 3.5 | 1989-1992 | -9.9 | 1992-2014 | 0.9* | 2014-2016 | -14.2 |  |  |  |  |
| Czech R. | -1.4* | 1986-2016 | -1.4* |  |  |  |  |  |  |  |  |  |  |
| Hungary | -1.5 | 1980-1986 | 3.1 | 1986-1994 | -3.3* | 1994-1997 | 6.6 | 1997-2016 | -3.4* |  |  |  |  |
| Montenegro | -19.0* | 2000-2009 | -19.0* |  |  |  |  |  |  |  |  |  |  |
| Poland | -1.0 | 1980-1983 | -1.9 | 1983-1987 | 6.3* | 1987-1993 | -2.3 | 1993-1996 | 2.4 | 1996-2012 | -1.7* | 2012-2015 | -6.5* |
| Romania | 4.3* | 1980-2016 | 4.3* |  |  |  |  |  |  |  |  |  |  |
| Serbia | -2.7* | 1998-2015 | -2.7* |  |  |  |  |  |  |  |  |  |  |
| Slovakia | -4.3* | 1992-1998 | -16.6* | 1998-2014 | 0.8 |  |  |  |  |  |  |  |  |
| Slovenia | -0.5 | 1985-1987 | -28.2 | 1987-2015 | 1.9* |  |  |  |  |  |  |  |  |
| TFYR Macedonia | 2.3* | 1991-2013 | 2.3* |  |  |  |  |  |  |  |  |  |  |
| **Eastern Europe** | **-1.6** |  |  |  |  |  |  |  |  |  |  |  |  |
| Belarus |  |  |  |  |  |  |  |  |  |  |  |  |  |
| Estonia | -2.2* | 1994-2015 | -2.2* |  |  |  |  |  |  |  |  |  |  |
| Latvia | -2.8* | 1996-2015 | -2.8* |  |  |  |  |  |  |  |  |  |  |
| Lithuania | -0.6 | 1993-2016 | -0.6 |  |  |  |  |  |  |  |  |  |  |
| Republic of Moldova | -1.0 | 1991-1995 | -17.1* | 1995-1998 | 54.4 | 1998-2016 | -4.4* |  |  |  |  |  |  |
| Russia |  |  |  |  |  |  |  |  |  |  |  |  |  |
| Ukraine |  |  |  |  |  |  |  |  |  |  |  |  |  |
| **Central Asia** | **-1.9** |  |  |  |  |  |  |  |  |  |  |  |  |
| Armenia | -5.3* | 2008-2014 | -9.3 | 2014-2016 | 7.6 |  |  |  |  |  |  |  |  |
| Azerbaijan |  |  |  |  |  |  |  |  |  |  |  |  |  |
| Georgia | 1.4 | 1998-2004 | -44.3 | 2004-2015 | 40.6* |  |  |  |  |  |  |  |  |
| Kazakhstan | -2.8 | 1991-1998 | 14.8* | 1998-2001 | -50.2 | 2001-2015 | 3.2 |  |  |  |  |  |  |
| Kyrgyzstan | -1.0 | 2000-2015 | -1.0 |  |  |  |  |  |  |  |  |  |  |
| Tajikistan |  |  |  |  |  |  |  |  |  |  |  |  |  |
| Turkmenistan |  |  |  |  |  |  |  |  |  |  |  |  |  |
| Uzbekistan |  |  |  |  |  |  |  |  |  |  |  |  |  |
| **Middle East and North Africa** |  |  |  |  |  |  |  |  |  |  |  |  |  |
| Turkey | 0.4 | 2009-2015 | 0.4 |  |  |  |  |  |  |  |  |  |  |

| **Colour** | **Average APC** | **Final segment only** |
| --- | --- | --- |
|  | Significant decrease | Significant decrease |
|  | - | Significant decrease but plateauing |
|  | No significant change | No significant change |
|  | Significant increase | Significant increase |
|  | - | No data available |

APC = Annual Percentage Change for one segment of a trend **Key:**

Average APC = Average APC for overall period

^a^AAPC for geographical regions = median values for constituent countries

*Rate of change significantly different from 0 at p<0.05
